# Danshen (*Salvia miltiorrhiza*) on the Global Market: What Are the Implications for Products’ Quality?

**DOI:** 10.3389/fphar.2021.621169

**Published:** 2021-04-26

**Authors:** Ka Yui Kum, Rainer Kirchhof, Rainer Luick, Michael Heinrich

**Affiliations:** ^1^ Pharmacognosy and Phytotherapy, UCL School of Pharmacy, London, United Kingdom; ^2^ University of Applied Sciences, Hochschule Rottenburg, Rottenburg am Neckar, Germany

**Keywords:** metabolomics (OMICS), H-NMR, HPTLC, ICP-OES (coupled plasma optical emission spectrometer), danshen (Salvia miltiorrhiza bunge.), quality assessement, MTT assay, griess assay

## Abstract

**Background:** Danshen (Radix et rhizoma *Salviae miltiorrhizae*; *Salvia miltiorrhiza* Bunge, Lamiaceae) is commonly used in Asia, including China, Japan, and Korea with markets in America and Europe growing substantially. It is included in multiple pharmacopeias and salvianolic acid B and tanshinone IIA are used as quality markers. However, on the markets, substitutes and different processing methods often are a concern. a concern regarding patients’ safety and expected outcomes.

**Aims:** This study aims at understanding the quality of Danshen-derived products on the market, and the relationship between the chemistry, biological activity and the processing and storage methods.

**Methods:** For heavy metal analysis, inductively coupled plasma optical emission spectrometry was used. High performance thin-layer chromatography and proton nuclear magnetic resonance coupled with principal component analysis were used to understand the variation of metabolite composition. MTT assay and LPS induced NO production assay were used to evaluate the cytotoxicity effect and anti-inflammatory activity, respectively.

**Result and Discussion:** Six out of sixty samples exceed the limits of cadmium according to the Chinese or United States Pharmacopoeia. Arsenic, lead and copper contents are all below pharmacopoeial thresholds. With more complex processing procedure, the risk of heavy metal contamination increases, especially with arsenic and cadmium. The metabolite compositions show a variability linked to processing and storage methods. Authenticated samples and Vietnamese primary samples contain higher salvianolic acid B, and their chemical compositions are more consistent compared to Chinese online store samples. Overall, a significant chemical variation can be observed in Danshen products directly linked to processing and storage method. In the MTT assay, fourteen samples show cytotoxicity while seven samples increase the proliferation of RAW264.7. In the LPS induced NO production of RAW 264.7, only seven samples show significant inhibitory effects.

**Conclusion:** This is the first interdisciplinary investigation focusing on understanding the current market and the quality of Danshen. The quality of Danshen products on the high street are inferior to the authenticated samples. The results of the bioassays selected is not useful to differentiate the quality and composition according to the current definition in the pharmacopoeias. Overall, this approach highlights the tremendous variability of the products linked to processing and the need for more systematic and stringent quality assurance.

## Introduction

Radix *et* rhizoma *Salviae miltiorrhizae* (Danshen, from *Salvia miltiorrhiza* Bunge, Lamiaceae) has been used commonly as a traditional Chinese medicine in many Asian countries such as China, Japan, and Korea. It is one of the most important botanical drugs in modern TCM. Based on traditional Chinese medicine theory, Danshen has the properties of removing blood stasis and promoting blood circulation; clearing menstruation and relieving pain and swelling (Zhou et al., 2005). Between 200 and 300 B.C., “Shen Nong’s Classic of the Materia Medica”—Shennong Ben Cao Jin (*Anonymous*) cited Danshen as a better-class medicine. In 2015, the total demand of Danshen was estimated to be 18.4 thousand tons according to a market report (Zhiyan Consulting Group, 2017).

The common medical indications for Danshen include menstrual disorders, cardiovascular diseases, chronic pain and thoracic obstruction. Currently, more than thirty clinical studies related to using Danshen to treat different cardiovascular diseases have been conducted, and they have shown promising evidence for improving the clinical symptoms (Wang et al., 2017a). Furthermore, some scientists study other potential medical indications of Danshen including diabetes (Jia et al., 2019), stroke (Bonaccini et al., 2015; Kim et al., 2018) and cancer (Chen et al., 2013; Wang et al., 2017b). The species is known well in terms of its chemistry. Until now, more than 40 abietane diterpenes (tanshinones) and 50 phenolic acids (salvianolic acids) have been identified (Jiang et al., 2005; Zhang et al., 2012; Chen et al., 2014). These are essential for understanding the botanical drug’s pharmacological activity.

In the current understanding, tanshinone IIA inhibits the oxidization of the low-density lipid oxidization in RAW 264.7 macrophage via NF-κβ signaling (Jang et al., 2006; Chen et al., 2007). The possible anti-inflammatory effect is linked to inhibiting the expression of IL-1, IL-6, and TNF-α in NF-κβ pathway (Jang et al., 2003) as well as moderating IKK, ERK, and JNK in MAPKs and Nrf2 pathway (Zhang and Wang, 2007; Li et al., 2010; Bi et al., 2012). Several studies show that salvianolic acid B also inhibits the oxidization of low-density lipids (Zhao et al., 2008; Ho and Hong, 2011; Yang et al., 2012) and protects vascular endothelial cells by regulating VCAM-1, ICAM-1, TNF-α, etc. (Du et al., 1995; Chen et al., 2001; Lay et al., 2003; Pan et al., 2011). These pathways are linked to NO production releases from different cells including macrophages and endothelial cells (Black and Garbutt, 2002; Berg and Scherer, 2005; Moore and Tabas, 2011; Mangge, 2014) and closely related to cardiovascular diseases.

Many pharmacopoeias have recognized Danshen (British Pharmacopoeia, 2015; Chinese Pharmacopoeia, 2015; Japanese pharmacopoeia, 2017; United States Pharmacopeial Convention, 2016). The quality of Danshen is defined on the basis of salvianolic acid B and tanshinone IIA as markers, but their standard levels vary in the different regulatory systems (Table 1). However, Pang et al., 2016 argues that the current quality control from pharmacopoeias using salvianolic acid B and tanshinone IIA is inadequate as more metabolites in Danshen, such as cryptotanshinone, rosmarinic acid and tanshinone I, are of importance for the pharmacological activities. Different phytochemical analytical methods for Danshen such as HPTLC (Hu et al., 2005), UV (Wang et al., 2014; Yan et al., 2016), MS (Liang et al., 2017; Ni et al., 2019) and NMR (Jiang et al., 2014) have been developed. The current quality standard of Danshen does reflect these the scientific advances.

**TABLE 1:** Comparison of Radix et Rhizoma Salvia miltiorrhiza quality criteria based on different regulatory bodies. Gray boxes–no quality parameter specified.

		ChP 2015	Thp 2nd	USP 40th	Jp 17th	Ph. Eur 9th	BP 2015	Hkcmms	WHO	ISO
Water-soluble extractives		50%	35%	35%				57%		
Alcohol-soluble extractives		46%	15%	15%	42%			52%		
Tanshinones		0.25%		0.20%						
Tanshinone IIA			0.20%	0.10%		0.12%	0.12%	0.12%		
Salvianolic acid B		3%		3%		3%	3%	4%		
Rosmarinic acid								0.17%		
Loss of drying		13%	15%	13%		10%	10%	12%		
Ash insoluble		10%	10%	10%		10%	10%	8%		
Ash insoluble in HCl		3%		3%		3%	3%	2%		
Heavy metal (mg/kg)	Pb	5	5	5	10	5	5	5	10	10
	Cd	0.3	0.3	0.3		0.2	1	1	0.3	4
	As	2	2	2	5		5	2		2
	Hg	0.2	0.2	0.2		0.1	0.1	0.2		3
	Cu	20	20							

In addition, the variability of Danshen materia prima available on the markets complicates attempts for consistent quality control. Historically, the production of Danshen was limited to Shandong, Henan, Hubei and Sichuan. One of the earliest investigations on tanshinones showed the tanshinone IIA contents varying from 0.02 to 0.32% depending on the origins. Seven out of ten samples contained under 0.12% tanshinone IIA, only the Danshen samples from Shandong (0.32%), Henan (0.23%) and Hubei (0.16%) had more than 0.12% (Huang et al., 1980a; Huang et al., 1980b). Today, dried roots are cultivated commercially all over China except in Hainan, Inner Mongolia, Jilin, Heilongjiang, Tibet and Xinjiang, but the processing and cultivation methods vary. Some countries in Asia, including Taiwan, Japan and Vietnam, have small production areas, but the majority of the Danshen used is imported.

Another report (Zhang et al., 2018b) states the substitute species and the increase of new cultivars extravagate the quality problem particular in Danshen. At least 19 other *Salvia* species have been used traditionally as Danshen (Xu et al., 2018) in some local areas, and of these, *S. bowleyana* Dunn. and *S*. *przewalskii* Maxim are the most common substitutes. Both have a chemical composition similar to *S. miltiorrhiza*, including salvianolic acids and tanshinones. It is common for farmers to cross-pollinate and cultivate better disease-resistant hybrid cultivars. Two cultivars, *S. miltiorriza* var. *miltiorrhiza* and var. *charbommellii*, are documented under the taxa of *S. miltiorrhiza* in the flora of China (Li and Ian, 2015). These practices impact on the quality of Danshen and it is difficult to identify these cultivars as they have similar chemical compositions.

In the 2015 reports of NMPA (Zhang et al., 2018a and b), 39.7% of Danshen (defined as Radix *S. miltiorrhiza*) did not pass the tests of macroscopic characteristics, the extract, water or biomarker content. It had one of the highest failure rates among of the TCM materials. This raises two issues: 1) What are the factors of the consistency of the quality in Danshen products? and 2) What is the definition of Danshen “quality” regarding safety and therapeutic effects? The current standard criteria of TCM uses major secondary metabolites or characteristic metabolites to define the quality. Nevertheless, quality is an essential basis for safety and potential bioactivities. Therefore, an integrated approach to chemical composition and bioactivity is needed (Wang et al., 2019).

## Aims and Objectives

Clearly, better ways for a consistent high-quality supply of the primary material and finished products are needed. Ways to ascertain this require an understanding of what problems actually cause poor quality. Moreover, there is a lack of understanding of the quality of Danshen derived products. Authenticity and quality assessment need to be understood as a consequence of an entire specific value chain. This project aims to understand the quality of plant derivative medicinal products using a phytochemical-metabolomic approach combined with a bioassay and an assessment of one class of potential contaminants—heavy metals.

## Materials and Methods

### Sample Collection

Authenticated *S. przewalskii* Maxim, *S. bowleyana* Dunn and *S. miltiorrhiza* Bunge samples were obtained from NIFDC, Kew Gardens, America Herbal Pharmacopoeia, Brion, Lfl and YuFu biotek. Authenticated samples were collected by botanists and were processed straight after the harvest.

Commercial samples were collected in Phố Lãn Ông (Vietnam) or on “Taobao” (a Chinese online store platform). The reason for examining Phố Lãn Ông is that the traceability was low and the quality of the herbal materials in Vietnam was not well regulated.

The biggest Chinese e-commerce platform Taobao which covering accredited online stores and typical online sellers was chosen as one of the channels of collecting samples. During May 2017, “丹参” (the simplified Chinese word of Danshen), and “丹参粉” (Danshen powder) were used as keywords.

All samples are deposited at the School of Pharmacy’s collection of commercial and pharmacognostic specimens, with the intention of a longer term integration into the collection of the Uebersee-Museum Bremen (BREM). The sample list with the sample information, such as origin, price, material form, is attached in the Supplementary Document.

### Sample Preparation

Three types of samples were collected: 1) crude dried roots or rhizomes, 2) dried root or rhizome powder and 3) concentrated extracts. The method of sample preparation was developed based on Kim et al. (2010). Crude roots or rhizomes samples were ground into fine powder by a mechanic grinder (GT203840, Tefal, United Kingdom), and passed through a sieve, resulting in particles of less than 1 mm size. All samples were stored with silica gel packs to avoid humidity.

All samples were accurately weighed with less than 0.5% error and extracted in 75% methanol with 1: 20 (g/ml) drug-solvent ratio followed by 30 s of vortexing and 30 min of ultra-sonication. The extracted samples were centrifuged under 1,400 rpm at 15°C for 10 min.

### Solvents

Milli-Q water (purified by Elix® S water purification with Q-Gard® 1 Purification Cartridge, Merck, Germany), chloroform, methanol and ethanol (HPLC grade, Sigma-Aldrich, United States), ethyl acetate, toluene and glacial acetic acid (HPLC grade, Fisher Scientific, United Kingdom), formic acid (ACS reagent, 98%, BDH Chemicals Ltd., United Kingdom), DMSOd6, ≥99.0% (Cambridge Isotope Laboratories, Inc., United States), PBS buffer pH 7.4, D-MEM, penicillin-streptomycin 10,000 U/ml, FBS and Trypan blue stain 0.4% (Gibco, Stockholm, Sweden), MTT ≥ 97.5% HPLC grade, DMSO anhydrous ≥99.9%, DMSO Hybri-Max® (Sigma-Aldrich, United States), H_2_O_2_ (HPLC grade, Sigma-Aldrich, United States/analytical grade, Carl Roth, Germany), HNO_3_ (analytical grade, Carl Roth, Germany), HCl (analytical grade, Carl Roth, Germany), phosphoric acid (≥98%, ACROS Organic, United States), RotiStar ICP-standard matrix: 5% HNO_3_ (Carl Roth, Germany).

### Cell Line

RAW 264.7 (American Type Culture Collection, United States).

### Chemical Standards

DSS (NMR grade, ≥99.9%, Sigma-Aldrich, United States), sulfanilamide ≥99%, N-1-naphthylethelene (ACS reagent, ≥98%, Sigma Aldrich, United States), MTT ≥ 97.5% HPLC grade, LPS from *Escherichia coli* (Sigma Aldrich, United States).

Salvianolic acid A ≥ 98%, salvianolic acid B ≥ 95%, danshensu ≥ 98%, rosmarinic acid ≥ 98%, caffeic acid ≥ 98%, tanshinone IIA ≥ 98%, tanshinone I ≥ 96%, cryptotanshinone ≥ 98%, and dihydrotanshinone I ≥ 96% (Tauto Biotech, China).

### Instruments

Grinder (GT203840, Tefal, United Kingdom), Rotamixer (Hook and Tucker Instruments Ltd., United Kingdom), Grant XB22 ultrasonic bath (Grant Instruments, United Kingdom), Centrifugator (Centrifuge 5804 R, Eppendorf, Germany), electronic balance (Sartorius CP64, Sartorius AG, Germany), Freeze dryer (ModulyoD Freeze Dryer, Thermo Fisher Scientific, United Kingdom), NMR tube (VWR international Ltd., United States), Bruker Advance 500 MHz spectrometer (Bruker, Germany), HPTLC plates silica gel 60 F 254 (Merck, Germany), Linomat 5 (CAMAG, Switzerland) coupled with a 100 μL syringe (CAMAG, Switzerland) and compressed air with 60–90 psi., Automatic Developing Chamber ADC 2 (CAMAG, Switzerland), TLC Visualizer (CAMAG, Switzerland) Microwell plate Nunclon 96 well (Thermo Scientific Nunc, United Kingdom), GalaxyB CO_2_ incubator (Scientific Laboratory Supplier Ltd., United Kingdom), water bath (LAUDA Aqualine AL 12, Germany), microscope (Olympus CK40 microscope, Japan), plate shaker (MS3 basic, IKA®, Germany) microplate reader (Infinite M200, Tecan, Switzerland), Multiwave Go (Anton Paar, Graz, Austria), ICP-OES SPECTROBLUE T1 (SPECTRO Analytical Instruments, Kleve, Germany).

### Software

MestreNova 12.0.1-20560 (Mestrelab Research S.L., Spain), SIMCA 14.1 (MKS Umetric AB, Switzerland), Excel 365 (Microsoft, United States), VisionCATS (CAMAG, Switzerland), Smart Analyser Vision February 5, 0937 (SPECTRO, Germany).

### High Performance Thin Layer Chromatography

The HPTLC method was developed from Booker et al. (2014) and the “Hong Kong Material Medica - Radix Salviae Miltiorrhizae Monograph” Chinese Medicine Division of the Department of Health (2005). The experiment aimed at understanding the chemical differences between samples using a rapid screening technique. 2 μL 75% methanol extract of each sample was loaded on the HPTLC plate, as well as chemical standards. The HPTLC was started after the developing solvent system (MePh: CHCl_3_: EtOAc: MeOH: FA = 2: 3: 4: 0.2: 2) saturated for 20 min and pre-conditioned for 5 min. The condition of the tank was held at the humidity of 33% and a temperature of 23°C. The process stopped once the developing solvent reached the plate to 80 mm migration distance. After 5 min plate drying, the plates were visualized under white light, 254 and 366 nm wavelength. VisionCATS, the HPTLC workflow and analytical software, was used in plate analysis and graphic editing.

### Heavy Metal Analysis

The method of heavy metal analysis was developed based on Cooper et al. (2007), Olesik et al. (1995). 300 mg of powdered sample was weighed accurately in the microwave digestion tube. 1 ml of H_2_O_2_ for 10 min, 2 ml of 65% HNO_3_ for an hour and another 2 ml of 65% HNO_3_ for an hour, and 1.5 ml of 35% HCl for 18 h were added to each sample. Before microwave digestion started, samples 7.5 ml of 35% HCl was added. The tubes were sealed and placed in the microwave digestion system. After digestion, the digestates were diluted into 50 ml milli-Q water and the samples were analyzed by ICP-OES. The programs of the microwave digestion and the operation system and the limitation of the detection were listed in Supplementary Document.

### Sample Preparation for Bioassay and ^1^H-NMR

The methanol content of the supernatant was evaporated at 60°C dry heat plate under a fume hood for 1.5 h and freeze-dried. All extracts were stored in −80°C freezers and defrosted as needed.

### Maintenance of RAW 264.7 Cell Line

The RAW 264.7 cell line, a macrophage transformed from Abelson murine leukemia virus, was cultured in D-MEM supplemented with 10% heat-inactivated FBS (Hyclone, Utah, Logan, United States), penicillin-streptomycin (100 IU/ml and 100 μg/ml). The cells were incubated in a humidified atmosphere at 37°C with 5% CO_2_ supplied. Only passages from 5 to 11 of RAW 264.7 were used.

### Cytotoxicity in RAW 264.7 Cells

A variant of the MTT assay developed from Funk et al. (1993) was used for evaluating cytotoxicity. It aimed at understanding the biological effect of extract to macrophage cells. 200 μL of RAW 264.7 was seeded at a density of 3 × 10^4^ cells/μl in 96-well plate. The cells were incubated at 37°C and 5% CO_2_ supplied. After 24 h, the cells were treated with the sample at the concentrations of 100, 50, and 25 μg/ml, as well as the blank and the negative control for 24 h. All treatments were adjusted to the final concentration of 0.05% DMSO. After treatment, the cells were incubated with 100 μL of the MTT solution, which was 5 mg/ml of MTT dissolved in PBS mixed with DMSO at the ratio of 1:10, for 4 h. After the removal of the MTT solution, 100 μL of DMSO was added into each well, and the plate was shaken horizontally for 1 min. Cell viability was measured at 570 nm using a microplate reader.

### Inhibition of LPS Induced NO Production

The Griess assay (Jin et al., 2009) aimed at understanding the anti-inflammatory activity of different Danshen products was used. 200 μL of RAW 264.7 was seeded at the density of 2.5 × 10^5^ cells/μl in the 96-well plate. The cells were incubated at 37°C and 5% CO_2_. After 24 h, the cells were treated with the sample at the concentrations of 100, 50, and 25 μg/ml, as well as the blank, negative control and positive control which was 20 μL indomethacin. All the treatments were adjusted to the final concentration of 0.5% DMSO. After the treatment, the cells were incubated with 50 ng/ml of the LPS for 24 h. Griess solution A and B (solution A: 4% sulfanilamide in 10% phosphoric acid; solution B: 0.4% N-1-naphthylethelene) were freshly prepared within an hour before the next step. 100 μL of cell culture medium was mixed with 25 μL of solution B, and 25 μL of solution A. 100, 50, 25, 12.5, 6.25, 3.125, 1.5625 μM NaNO_2_ and non-phenol red D-MEM were used as the reference standard of NO_2_^−^. The reaction was undertaken under light-protection, and the plate was shaken horizontally for 1 min after. The absorbance was measured at 550 nm wavelength in the microplate reader. The calculation of nitrate/nitrite concentration was calculated as follows:

$$\text{Nitrate}/\text{nitrite}\;\text{concentration}=\;\frac{\left[\text{Ab}-{\text{Ab}}_{\text{blank}}\right]}{\text{Slope}\;\text{of}\;{\text{NaNO}}_{2}\;\text{standard}\;\text{curve }},$$

whereas Ab, the absorbance of the well with cells with different treatment, Ab_blank_, absorbance of the well with complete medium without seeding cells.

Each sample was run in three replicates in a plate with three repeats (*n* = 9). All results were expressed as with means ± SEM. Data were analyzed using Student’s t-test using Excel 365. *p* < 0.05 was considered significant.

### Nuclear Magnetic Resonance and Metabolomic Profiling

The method for NMR analysis was developed from Li et al. (2015). This experiment aimed at understanding the chemical variation of market samples and differentiate the samples using multivariate analysis. 275 μL 200 mg/ml of each sample dissolved in DMSO_d6_ was mixed with 275 μL of 0.2% DSS dissolved in DMSO_d6_. All the samples were analyzed by 500 MHz ^1^H-NMR spectrometry with 256 scans which required 20 min approximately. The parameters of ^1^H-NMR spectra were listed below:

Spectral width = 10,330.578 Hz, 0.1576 Hz per point, pulse width = 13.9 μsec and relaxation delay (RD) = 1.0 s. Free induction decays (FIDs) were Fourier transformed with a line broadening (LB) of 0.3 Hz. All the sample were repeated twice at least individually.

The NMR spectra were processed by the analytical chemistry software, MestreNova. All the spectra were stacked and optimized with the interactive phase correction in global metabolomics mode, and the baseline correction with Bernstein polynomial fit order = 3. The chemical shift of DSS, set at 0 ppm, was used as the internal standard and normalized all the spectra. The range from δ 9.00 ppm to δ 0.66 ppm of the spectra were selected and the following areas were manually cut: δ 5.5 to δ 3.15 ppm, δ 2.6 to δ 2.4 ppm, δ 2.02 to δ 1.99 ppm, δ 1.70 to δ 1.50 ppm, and δ 1.25 to δ 1.16 ppm. The spectra were binned into 0.03 ppm by the method of the average sum. All data were analyzed by the multivariate analysis software, SIMCA.

### Multivariate Analysis (PCA)

All data, such as product information, NMR binned spectra, cytotoxicity MTT and LPS induced NO inhibition, was input to the dataset of SIMCA software. NMR binned spectra data was set as X variables, whereas other data was set as Y variables. The data were scaled by Pareto scaling. PCA-X was performed to evaluate the chemical variation level between samples. The PLS-DA was performed to evaluate the interrelationship between the chemistry and other information of the samples including the pharmacological evidence. The R^2^ and Q^2^ were used to understand the goodness of fit and predictability of the model. The first two components were used, and loading plot and VIP were used to evaluate the importance of the X variables to the model and sample distribution. The outliers among the samples were identified using the hotelling T_2_ range. HCA was used in categorizing samples with a similar pattern.

## Results and Discussion

In order to understand the quality issues, we used a range of established and non-standard techniques. As a baseline analysis, we looked at the heavy metal levels and the content of biomarker using HPTLC as described in the Chinese Pharmacopoeia. HPTLC and heavy metal analysis are the typical quality evaluation of herbal medicine. However, the content of heavy metals does not have therapeutic effects, and HPTLC does not provide a full spectrum of chemical composition. Hence, in addition, two more broad-spectrum methods ^1^H-NMR profiling and an *in vitro* test were employed. It will help to understand the suitability of identifying quality marker using these models.

### The Difference Between Samples Collected From Different Channels

Seventy-one samples were studied and information on geographic origin, additives and processing method was collected where possible. The samples from Chinese online stores have most of the product information listed on the website, but the physical store retailers can only provide a general geographical origin of their sample. It implies that physical retailers do not pay adequate attention to product information, and it shows low traceability of Danshen raw materials.

Of the fifteen samples sourced in Vietnam, four samples could not be traced to a geographical origin due to the lack of information provided by the retailer. Another four samples were cultivated in Vietnam while seven samples were imported from China. According to the field observation on the Vietnamese herbal market and the communication with the retailers, they weekly sundry their Danshen dried roots. This processing of Danshen materials is not often seen in other countries.

Chinese online store samples represent typical processing and storage method in China. They are generally dried in bulk size, and the retailers stored the materials under cool and dry condition. Authenticated samples are processed in a standardized operation. The roots were harvested carefully to avoid harming the materials and dried immediately after harvest. The dried materials are kept in a sealed, cool and dry condition.

### Heavy Metal Levels in Danshen Derived Products

**FIGURE 1:**
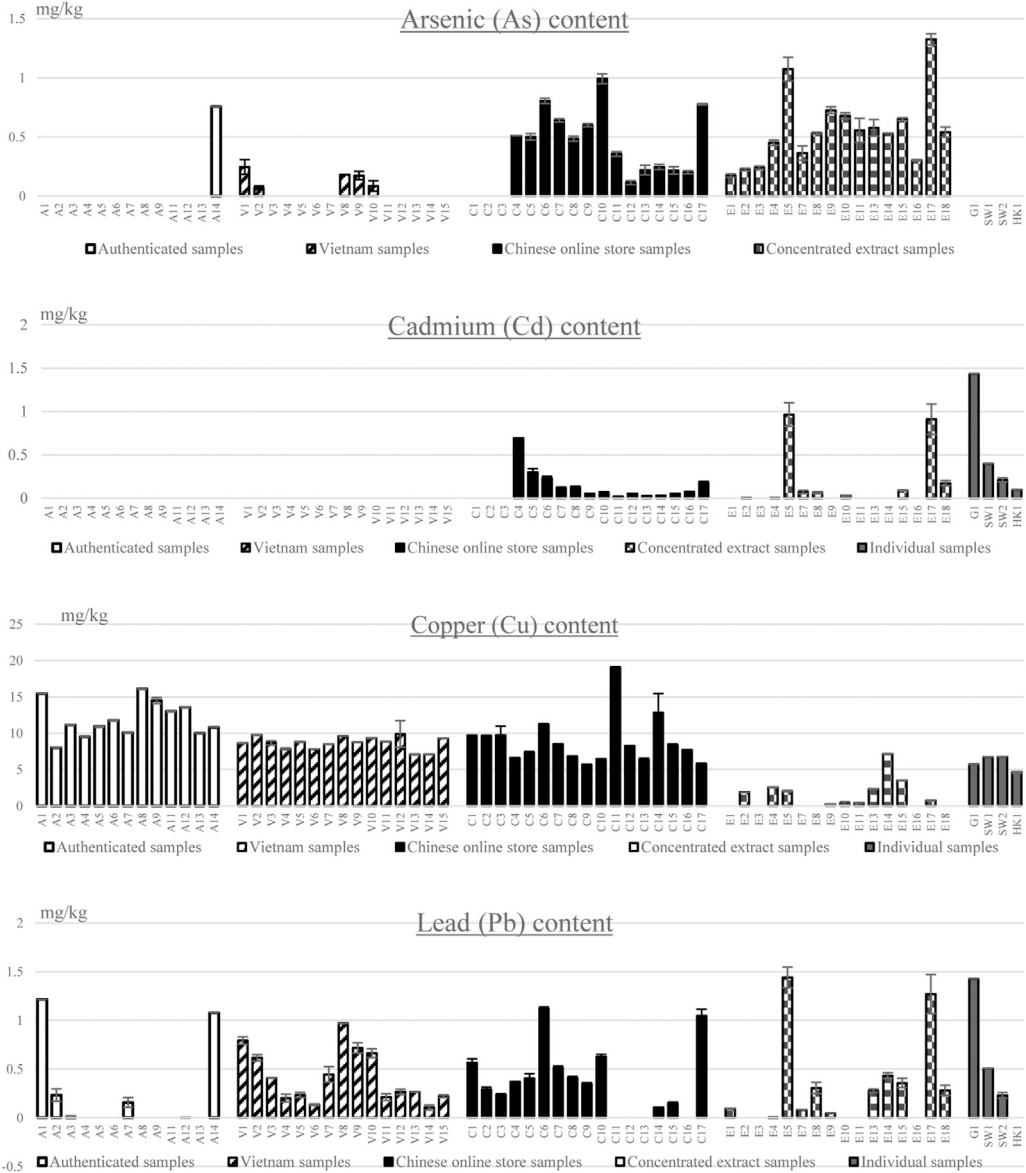
Heavy metal analysis of authenticated samples (A1–A14), Vietnamese samples (V1–V15), Chinese online stores samples (C1–C17) and concentrated extracts (E1–E5, E7–E11, and E13–E18) as well as some individual samples (G1, SW1, SW2, and HK1).

In the heavy metal analysis (Figure 1), none of the samples exceeds the limits of As, Cu or Pb as set in any pharmacopoeia. All samples are below the limits for arsenic based on the ChP or other pharmacopoeias (less than 2 mg/kg). Only six samples from authenticated, Vietnamese and individual samples obtain detectable arsenic content and most of them are lower than 0.2 mg/kg. The highest As content is from E17 (a concentrated extract), which has 1.33 mg/kg. The lowest limit of lead among the pharmacopoeia is 5 mg/kg, and all samples are under 1.5 mg/kg (Figure 1).

The sample with the highest copper content (C11 with 19 mg/kg) is just under the 20 mg/kg limit of pharmacopoeias. The average copper content of the raw materials and the powdered material samples (9.64 mg/kg) are at least eight times higher than of the concentrated extracts (1.12 mg/kg) (Figure 1). One of the possible reasons is that the industrial extraction process of commercial *S. miltiorrhiza* extract would not dissolve all the copper in the roots of Danshen, but the heavy metal analysis extraction completely digests the materials. As the concentrated extracts are secondary products, the content of copper will be lower than raw materials. However, it needs further investigation to conclude.

However, six out of sixty samples exceed cadmium limits according to ChP or USP, including four commercial raw materials and two concentrated extracts (Figure 1). None of these samples is sourced from Vietnam or authenticated samples, and most of these show undetectable levels. Our result also shows that the more processing of the product has, the higher possibility of heavy metal contamination, especially in arsenic and cadmium.

To summarize, the results from ICP-OSE reflect that *S. miltiorrhiza* derived products do not have a severe excess of heavy metals. It is generally safe but with the occasional exception of cadmium. It also shows that the chance of heavy metal contamination increases with the increase of processing procedure. The study from Yan et al., 2012 shows a similar result in Cd level, but it also stated the excessive Cu in *S. miltiorrhiza* should be common. Furthermore, the study shows that heavy metal content does not have a strong relationship with its soil quality. However, not many heavy metal studies in *S. miltiorrhiza* products regarding the relationship between cultivation and processing methods have been carried out.

### Chemical Composition Variation of Danshen Products on the Market

To understand the variation of quality in terms of chemical composition in Danshen products, HPTLC and ^1^H-NMR-PCA were used. HPTLC is a typical analytical method in pharmacopoeias that is quick, easily accessed, and reasonably priced. However, as it is a chromatographic technique, the result depends on the combination of the stationary phase and the mobile phase. Hence it is not easy to obtain the whole spectrum of metabolites in the samples, for instance, polysaccharides.

**FIGURE 2:**
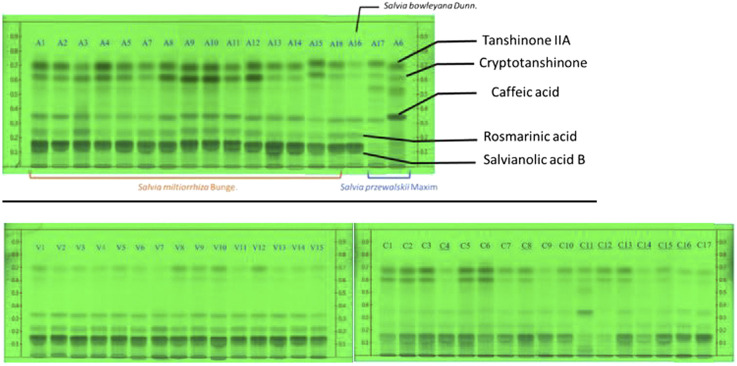
HPTLC results of authenticated samples, Vietnamese samples and Chinese online store samples. The HPTLC is performed under 254 nm developed with solvent system (toluene: chloroform: ethyl acetate: methanol: formic acid (v/v) = 2: 3: 4: 0.2: 2). A1–A18 are authenticated samples including *S. miltiorrhiza*, *S. bowleyana*, and *S. przewalski*. V1–V15 are Vietnamese samples and C1–C17 are Chinese online store samples. The samples underlined were sold as grounded powder while others were dried roots.

In the result of HPTLC (Figure 2), the authenticated samples have a higher level of major secondary metabolites of *S. miltiorrhiza*, for instance, salvianolic acid A, B, rosmarinic acid, caffeic acid, tanshinone IIA, cryptotanshinone and dihydrotanshinone. The samples of A8 to A12 come from the same source in Taiwan but are dried using different processing methods. The contents of salvianolic acids in these samples are similar, but A9, A10, and A12 have recognizably higher levels of tanshinone IIA and crytotanshinone in HPTLC. Compared to A11, they were dried at low temperature after cutting. A8 was dried at high temperature to imitate the farmers’ practice during the bad weather. It indicates that the degradation of secondary metabolites in *S. miltiorrhiza* relates to water retention time, drying temperature and cutting.

In addition, *S. bowleyana* and *S. przewalskii* are both distinct from authenticated *S. miltiorrhiza* in regard to the ratio of metabolites. *S. bowleyana* (A16) has very similar hydrophilic metabolites such as caffeic acid, salvianolic acid B and rosmarinic acid, but the contents of tanshinones are lower than authenticated *S. miltiorrhiza* samples in general. On the other hand, *S. przewalskii* (A6 and A17) has more tanshinones, including tanshinone IIA cryptotanshinone and perhaps other tanshinones compared to *S. miltiorrhiza* samples. The contents of salvianolic acid B and rosmarinic acid are merely undetectable, however, one of the *S. przewalskii* samples (A6) has an exceptionally high level of caffeic acid. This result matches with the results from other studies on *S. bowleyana* and *S. przewalskii* (Huang et al., 1980b; Li et al., 1993).

Vietnamese samples also show high levels of salvianolic acid B and slightly lower levels of caffeic acid and rosmarinic acid (Figure 2). However, the tanshinone levels in Vietnamese samples are significantly lower than the authenticated samples. The chemical compositions of Vietnamese samples are relatively consistent. These samples were sourced from a farm in Northern Vietnam and Chinese supplier(s), which include at least two different sources, this consistent result of HPTLC may be due to a similar supply chain, including processing and storage.

The HPTLC results of Chinese online store samples show that the qualities for these commercial products are highly diverse, and there is a chance of adulteration in particular in the case of C11 (Figure 2). C11 has a high level of caffeic acid and some prominent bands on tanshinone IIA and other tanshinones, but it has a low level of salvianolic acid B and rosmarinic acid. This pattern is similar to A17 and A6, which are authenticated *S. przewalski* Maxim. C12 and C14 also have a low level of salvianolic acid B, which are possible to be poor quality or adulteration as well (Figure 2).

On the other hand, ^1^H-NMR is a comprehensive chemical analysis, but less sensitive compared to HPTLC. It identifies and quantifies the shield of hydrogens which can be applied to most of the organic compounds. Hence, it helps to compensate for the disadvantages of HPTLC adding more information, such as structures, chemical class, amount of chemicals and more, of the metabolites in the samples.

**FIGURE 3:**
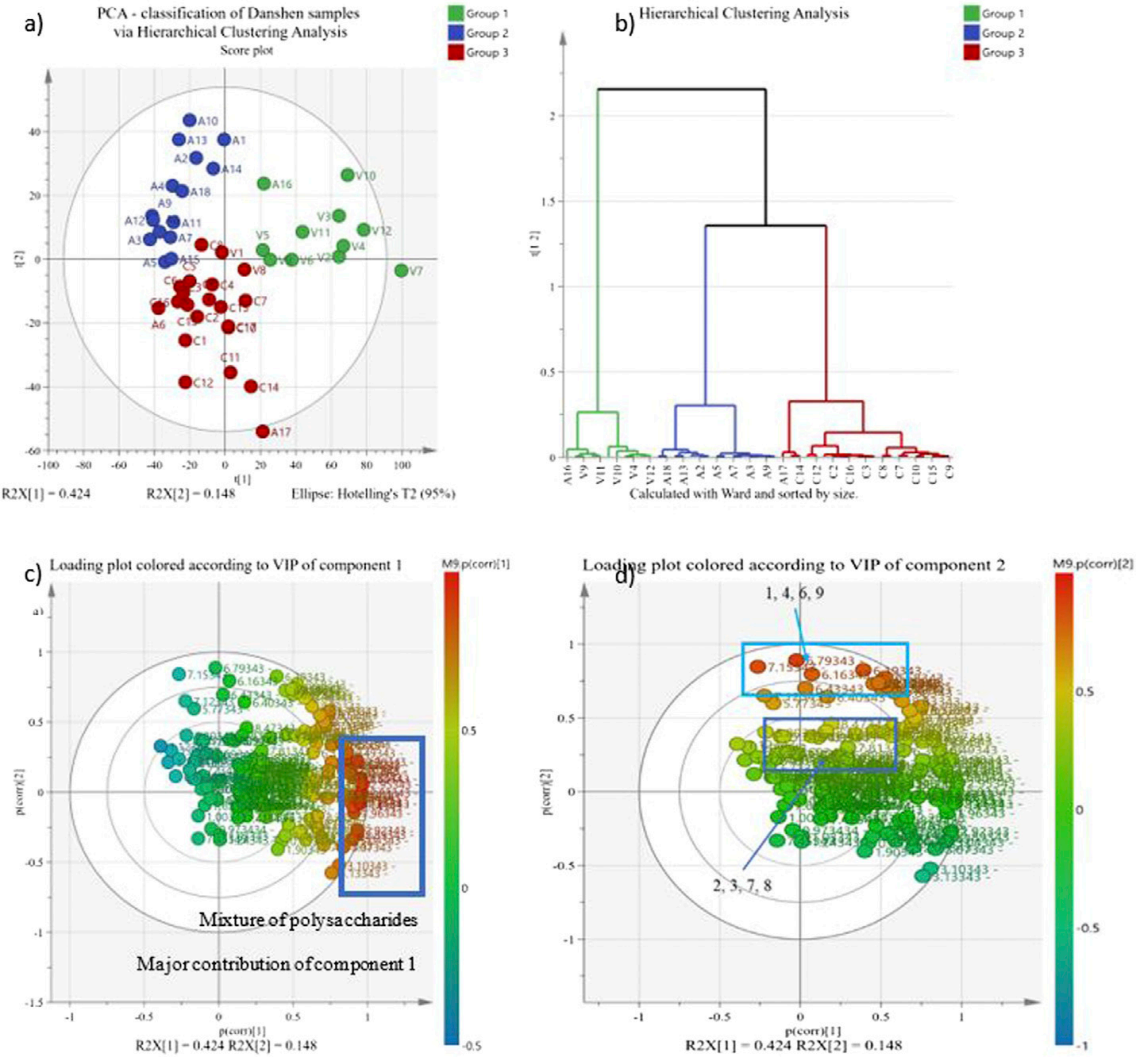
PCA of all Danshen samples. The PCA-X corresponds to the binned regions of all ^1^H-NMR spectra of Danshen samples as X variables. All X variables are scaled by Pareto scaling. In **(A)** PCA scatter score plot and **(B)** Hierarchical Clustering Analysis for PCA, Figure **(C)** and **(D)** are the loading plots of figure **(A)**. The colours of the loading plots are referring the p value of the X variable contribute to the principal component 1 in figure **(C)**, and the component 2 in figure **(D)**. A, authenticated samples; C, Chinese online store samples; V, Vietnamese samples

The ^1^H-NMR results are not quantified as the most of the R^2^ > 0.9 but not >0.997, resulting in a poor predictability in quantitative terms. The ^1^H-NMR-PCA result shows that the best correlation with the chemical variation is the sales channels (Figure 3). The estimate of goodness of fit (R^2^) and the estimate of goodness of prediction (Q^2^) are 0.572 and 0.49, respectively. The R^2^ and Q^2^ of an acceptable biological metabolomics model should be more than 0.4, but there are no fixed definitions of a good model (Broadhurst et al., 2006; Worley and Powers, 2013). Figure 3A demonstrates the samples’ chemical similarity according to ^1^H-NMR results. R^2^ and Q^2^ are expected to be smaller than other metabolomics models as the model has a high level of diversity in the sample collection. Hierarchical Clustering Analysis (HCA) is used to explore the classification of samples via the similarity of their chemical compositions and shown in Figure 3B.

All authenticated *S. miltiorrhiza* samples are classified in group 1 including samples cultivated in America (A1–A5), Germany (A13 and A14), Taiwan (A8–A12) and China (A7, A15, and A18). Apart from V1 and V8 (samples originated from China), the Vietnamese samples with unknown geographical origin (V4, V6, V14, and V15), imported from China (V5, V7, V10, V11, and V13) and cultivated in Vietnam (V2, V3, V9, and V12) are classified in group 2 as well as A16 (*S. bowleyana*), which indicates they have similar chemical composition. All the Chinese online store samples are categorized as group 3 with A6 and A17 (*S. przewalskii*) as well as V1 and V8. According to the HCA and PCA results in Figure 3, the authenticated *S. miltiorrhiza* samples from China are differentiated from Vietnamese samples imported from China or Chinese online store samples. Also, the authenticated *S. miltiorrhiza* samples from different geographical origins show high level of similarity in their chemical composition. Hence, the geographical origin does not show a direct relationship with the chemical composition in the PCA.

On the other hand, the HCA categorization highly matches with the sale channels and the processing and storage method of the samples. As mentioned, the samples group 1 are all authenticated *S. miltiorrhiza* samples and are processed and storage with a standard operation. The samples in group 2 are all from Vietnamese herbal market which the samples were sundried regularly. The samples in group 3 are either *S. przewalskii* or represent typical processing and storage method in the market. Hence, it is highly possible that there is a direct or indirect relationship between the chemical composition of Danshen samples and their processing and storage condition.

Figures 3C,D are the same model but show the VIP difference of component 1 and 2. VIP determines the contribution of plots, which are chemical shifts here, to the model. Figure 3C shows that principal component 1 differentiates samples by polysaccharides and Figure 3D shows that principal component 2 differentiates samples by chemical standards 1, 4, 6, and 9 which are salvianolic acid B, A, rosmarinic acid and caffeic acid respectively. All authenticated *S. miltiorrhiza* and Vietnamese samples are situated at the positive part of component 2, which has a high contribution from caffeic acid, salvianolic acid B and rosmarinic acid NMR binned region compared to Chinese online store samples.

**FIGURE 4:**
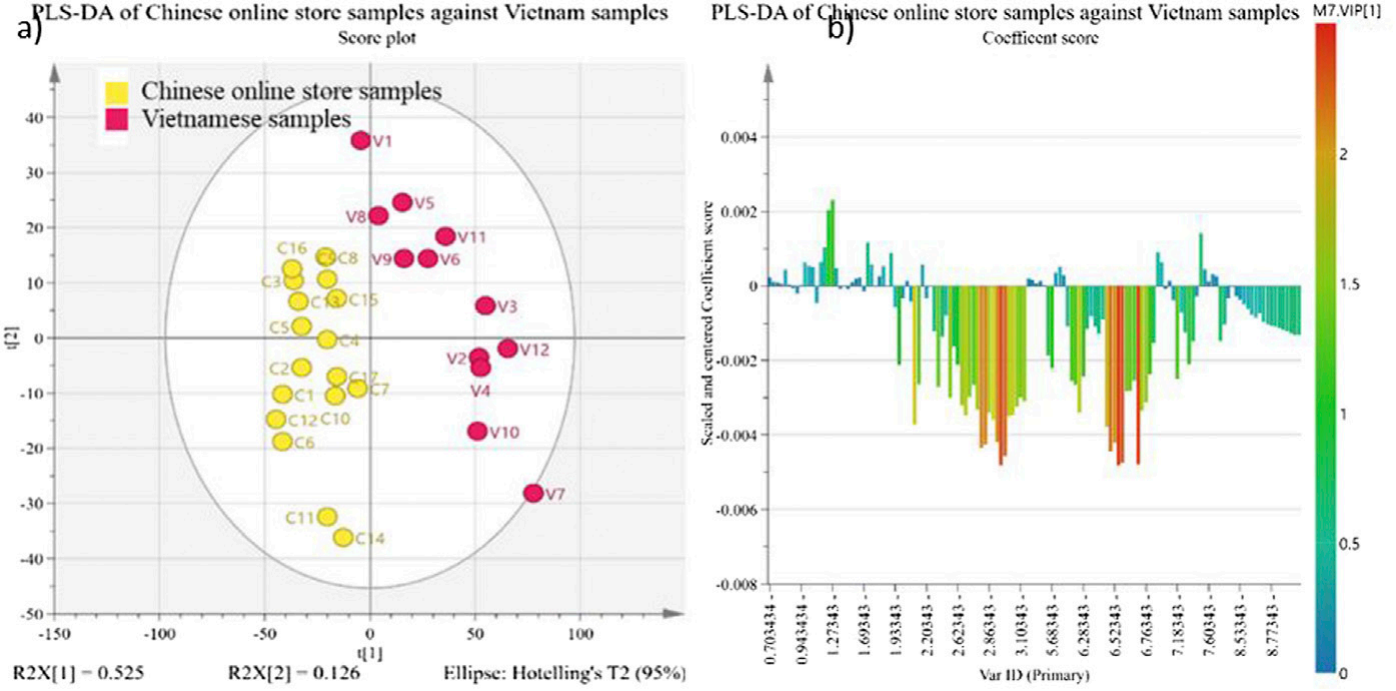
PLS-DA of Chinese online store samples against Vietnamese samples. The PLS-DA corresponds to the binned regions of ^1^H-NMR spectra of Chinese online store samples (C1–C17) and Vietnamese samples (V1–V12) as X variables and the value chains of samples as the Y variable. All X variables are scaled by Pareto scaling. The yellow colour in **(A)** PLS-DA scatter score plot represents the Chinese online store samples, and the pink represents Vietnamese samples. The colours of the samples in figure **(B)** loading plot are referring the VIP value of the X variable contribute to the principal component 1.

**FIGURE 5:**
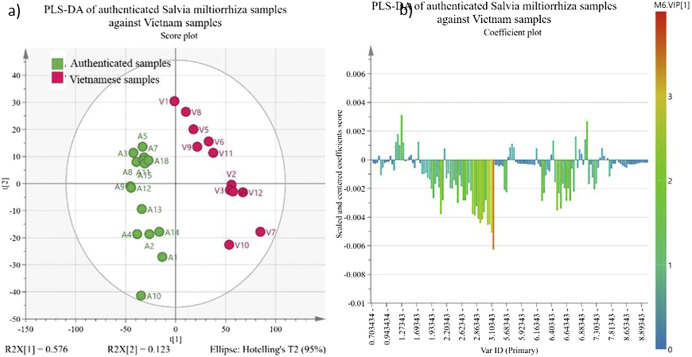
PLS-DA of authenticated *S. miltiorrhiza* samples against Vietnamese samples. The PLS-DA corresponds to the binned regions of ^1^H-NMR spectra of authenticated Salvia miltiorrhiza samples (A1–A18, except A6, A16, and A17) and Vietnamese samples (V1–V12) as X variables and the sales channel of samples as the Y variable. All X variables are scaled by Pareto scaling. The green colour in **(A)** PLS-DA scatter score plot and represents the authenticated Salvia miltiorrhiza samples and the pink represents Vietnamese samples. The colours of the samples in figure **(B)** loading plot are referring the VIP value of the X variable contribute to principal component 1.

While the PCA in Figure 3 is a general categorization statistical analysis, and the PLS-DA in Figures 4, 5 further investigates the chemical difference among samples. Figure 4 shows the chemical composition difference between Chinese online store samples and Vietnamese samples. The cumulative R^2^X, cumulative R^2^Y and Q^2^Y of this PLS-DA are 0.652, 0.86, and 0.78, respectively, and the permutation test shows a huge distinction between the random and actual model. These show the model is valid and good in fitness and prediction. Only one sample (V7) is the outlier of this model which means this sample is independent, and no samples are similar to it. Figure 4B shows that the Chinese online store samples have a higher contribution from tanshinone IIA, cryptotanshinone, tanshinone I and dihydrotanshinone I regions (δ 1.30–1.27 ppm and *δ* 7.6–7.0 ppm). Vietnamese samples have a higher contribution from salvianolic acids region (δ 6.8–6.0 ppm) and the primary metabolite region (δ 3.1–2.5 ppm), and it is significant to the model.

Similarly, Figure 5 shows the PLS-DA of the NMR results of authenticated samples against Vietnamese samples. The cumulative R^2^X, cumulative R^2^Y and Q^2^Y are 0.699, 0.903, and 0.867 respectively, and the permutation test shows a considerable distinction between the random and actual model. No samples are the outlier according to the hotelling T2 range. The authenticated samples have a higher contribution from tanshinone IIA, cryptotanshinone, tanshinone I and dihydrotanshinone I regions (δ 1.30–1.27 ppm and δ 7.6–7.0 ppm). Vietnamese samples have a higher contribution from the salvianolic acids region (δ 6.8–6.0 ppm) and the primary metabolite region (δ 3.1–2.5 ppm). Also, the region of δ 3.1–2.5 ppm contributes the most to the model.

Overall, these PCA and PLS-DA models demonstrate that Vietnamese samples have higher levels of salvianolic acids than authenticated samples than Chinese online store samples. Authenticated samples have higher tanshinones than Chinese online store samples than Vietnamese samples. Vietnamese samples also have significantly stronger signals in the primary metabolite region (δ 3.1–2.5 ppm). The PCA model also shows that Vietnamese samples and authenticated samples differ from Chinese online store samples. It indicates that the processing method and preservation are more critical to the chemical composition of *S. miltiorrhiza* derived products than geographical origin.

### Can a Bioassay Be Used to Assess the Quality of Danshen Preparations?

The MTT assay is widely used to understand the cytotoxicity and proliferative effects of samples. Here is employed to understand the variation in bioactivity safety, and it is a basis for conducting the Griess Assay. This LPS induced NO assay provides data on the anti-inflammatory activity of the samples, relevant since Danshen is used for pain-related and inflammatory vascular diseases. The activity of Danshen is closely related to NO signaling and NF-κβ pathway. These two assays aim to understand the bioactivity of different Danshen samples and whether it is possible to differentiate the quality of Danshen. Supplementary Table S6 shows the samples with cytotoxic effects and proliferative effect to RAW 264.7. The cytotoxicity activities of all samples are attached in the Supplementary Document.

In the MTT assay, twenty-one out of fifty-six samples tested shows considerable differences to the control group after applying Danshen sample extract at the initial concentration of 100 μg/ml. Fourteen samples show cytotoxic activity on the cells, including seven authenticated samples, five Chinese online store samples, and two Vietnamese samples. Apart from C4, the effects of these samples on the cells show a dose-response relationship. While A3 and A10 have the highest level of salvianolic acid B and tanshinone IIA, the cell viabilities of A3 and A10 drop to less than one fourth compared to the control. In general, there is a considerable variation in the level of activity of the different samples.

As mentioned, the samples of A8 to A12 come from the same source but were processed differently, showing the extreme differences among samples. A9 and A10 show the cytotoxicity, while A8 shows the increase of cell viability. Also, A8, which shows proliferation in cell viability, is dried in an oven at high temperature while A9 and A10, which are dried in an oven at low temperature and freeze-dried, respectively, show cytotoxicity in RAW 264.7. As expected, different processing results in changes to the biological activity.

On the other hand, seven samples show increases of cell viability, including four authenticated samples, one Vietnamese sample, one Swiss sample, and one Danshen concentrated extract. Of these, A7, A13, and A14 show an increase of more than 50% compared to the control. A13 and A14 are cultivated on the same farm with the same method and processed in the same way but in a different year. The time difference here does not significantly change the biological activities of the product, but further study needs to be done, for instance, more systematic sample collection and recording weather changes and soil condition.

The high level of bioactivity variation in MTT assay shows the bioactivity of Danshen varies from sample to sample and no specific pattern or relationship is found in the samples’ cytotoxicity or proliferation activity using PCA. Interestingly, Danshen samples on the market can lead both to cytotoxicity and proliferation on RAW 264.7 and all the samples show dose-response relationship expect C4, A8, and A13.

**FIGURE 6:**
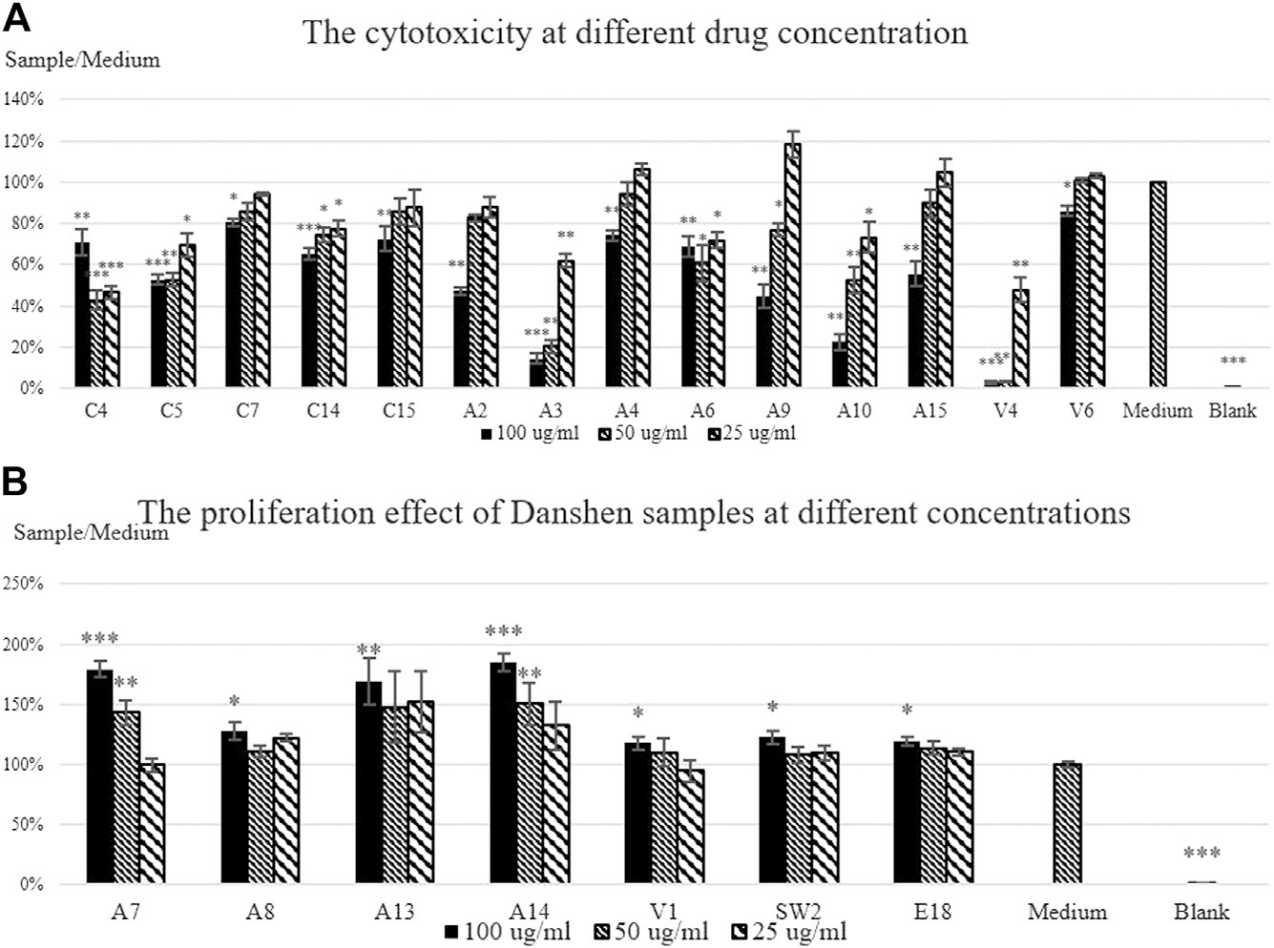
The cell viability results of Danshen sample extracts in RAW 264.7. 200 μl of RAW 264.7 with the density of 3 × 10^4^ cells/μl is treated with 100, 50, and 25 μg/ml of sample for 24 h. **(A)** the results of the samples showing the significant cytotoxic effect to RAW 264.7 **(B)** the results of the samples showing a proliferative effect in RAW 264.7. 0.05% DMSO was used as the control of the experiment. The cell viability percentage is the mean absorbance of the sample absorbance divided by the medium absorbance ± SEM of three triplicated independent experiments. Statistical significance of the sample is shown as * = *p* <0.05, ** = *p* <0.01, and *** = *p* <0.001 compared with the medium control.

**FIGURE 7:**
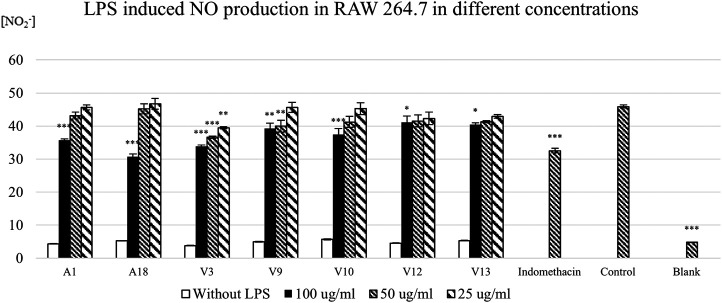
The effect of Danshen extracts on LPS induced NO production in RAW 264.7. 200 μl of the RAW 264.7 cells with the density of 2.5 × 10^5^ cells/μl is treated with 100, 50, and 25 μg/ml of sample for 24 h. 20 μg/ml of indomethacin is used as the positive control. The results of the samples that show significant inhibition on LPS induced NO production on the RAW 264.7. 0.05% DMSO was used as the control of the experiment. The NO concentration is based on the standard curve of NaNO_2_ ± SEM of three triplicated independent experiments. Statistical significance of the sample is shown as * = *p* <0.05, ** = *p* <0.01, and *** = *p* <0.001 compared with the medium control.

In the Griess assay, LPS triggers immunoresponse which increases the NO level; hence the model indicates the anti-inflammatory activity. Taken together differences in cell viability of RAW 264.7 after treatment with Danshen (Figure 6) in the LPS induced NO production (Figure 7; after exclusion of samples with cytotoxicity in RAW 264.7) demonstrate the variability in the biological effects of this set of Danshen samples. Within the non-cytotoxic Danshen samples, only seven samples, including two authenticated samples and five Vietnamese samples, show significant inhibitory dose-response dependent effects on the LPS induced NO production of RAW 264.7. Very few samples show anti-inflammatory effects in the Griess assay indicating that the chemical variation on the market significantly affects the biological activities of herbal products. V2, V9, and V12 are claimed to be cultivated in Vietnam, and two of them show NO inhibitions while all the Chinese online store samples do not inhibit NO signals. The anti-inflammatory activity of Vietnamese samples may also be linked to the storage practice of Danshen materials in the Vietnamese TCM herbal pharmacies.

Clearly, in the case of Danshen and the use of these bioassays, there is a correlation between processing and their bioactivities, but these assays are not useful to differentiate the quality and composition according to the current definition in the pharmacopoeia. Most likely, this is linked to the Danshen containing at least two classes of metabolites, which contribute to the activity and to the lack of comprehensive phytochemical profiling, with some minor compounds potentially having a substantial effect on these pharmacological targets. Nonetheless, in the standard of pharmacopoeia, a lot of samples are not identified as “poor quality” in chemical composition. Nevertheless, not many samples suggest anti-inflammatory activity and fourteen samples show a potential cytotoxicity to RAW 264.7 which may raise safety and quality issues. It indicates that the current chemical standards of *S. miltiorrhiza* in most of the pharmacopoeias are inadequate to assess biological activities of the product and to a certain extent, the quality as well.

## Conclusion

This study focuses on understanding the correlation of the phytochemistry, biological activity as well as the processing and storage methods of Danshen (*S. miltiorrhiza* Bunge.). It is also the first interdisciplinary study to evaluate the possibility of using trace metals analysis, HPTLC, ^1^H-NMR, with a combination of bioassays such as cytotoxicity and anti-inflammatory activity in a metabolomic approach. Clearly other quality parameters could have been included like the concentration of herbicides and pesticides, but this was not feasible within this project. This study also compares different authenticated samples from different geographical origins as well as marketed samples. It reveals a detailed picture of Danshen and its derivatives’ quality.

This study demonstrates that the quality of Danshen varies among different sales channels. No major problems were identified with regards to heavy metal content, but the variability, especially of the processed material, remains a problem. The variation in heavy metal content and the chemical compositions are directly linked to the processing of the product but not the geographical origin. The bioassay approach selected clearly resulted in ambivalent results. As the levels of the current chemical standards of Danshen are not the primary factors of the cytotoxicity and anti-inflammatory effects, it implies that the current chemical standards in pharmacopoeia do not represent the entire picture of the anti-inflammatory activity in Danshen. A future option would be to explore other pharmacological *in vitro* assays especially ones associated with potential cardiovascular targets.

It is evident that the Danshen products on the market show huge variations in chemical composition and biological activities. The chemical compositions of the authenticated samples and Vietnamese samples from the early stages of a value chain are more consistent than Chinese online store samples. This is similar to previous findings from our group, e.g. on St. John’s wort and roseroot (Booker et al., 2016; Scotti et al., 2019) where in general, the main chemical variability was shown to be due to differences in the value chains of the products. It shows that it is worth investigating the possibility of cultivating herbal materials globally combined with an appropriate and sustainable processing method, instead of cultivating in the “traditionally authenticated” herbal materials region. Further investigations using a metabolomics approach to understand the relationship between phytochemistry and biological activity are needed.

## Data Availability

The raw data supporting the conclusions of this article will be made available by the authors, without undue reservation.

## Abbreviations

°C, degree Celsius; ; 1H-NMR, proton nuclear magnetic resonance; As, arsenic; BP, British pharmacopoeia; Cd, cadmium; CFDA, china food and drug administration; CHCl3, chloroform; ChP, Pharmacopoeia of the people’s republic of China; CNS, central nervous system; Cu, copper; D-MEM, Dulbecco’s modified Eagle’s medium; DSS, sodium trimethylsilylpropanesulfonate; EtOAc, ethyl Acetate; FA, formid acid; FBS, fetal bovine serum; FDA, food and drug administration; HCA, hierarchical cluster analysis; HKCMMS, Hong Kong Chinese materia medica standards; HPLC, high performance liquid chromatography; HPTLC, high performance thin layer chromatography; ICP-OES, inductively coupled plasma—optical emission spectrometry; IL, interleukin; ISO, international organization for standardization; JP, Japanese pharmacopoeia; LPS, lipopolysaccharide; MeOH, methanol; MePh, toluene; MHRA, medicines and healthcare products regulatory agency; MMP, matrix metalloproteinase; MS, mass spectrometry; MTT, thiazolyl blue tetrazolium bromide; NF-κB, nuclear factor kappa-light-chain-enhancer of activated B cells; NIFDC, national institute of food and drug center; NMPA, national medical products administration; NO, nitric oxide; Par, Pareto scaling; Pb, lead; PBS, phosphate buffer saline; PCA, principal component analysis; Ph. Eur, european pharmacopoeia; PLS-DA, partial least squares discriminant analysis; Q^2^, the estimate of goodness of prediction; ^R^2^^, the estimate of goodness of fit; Rf, retardation factor; rpm, round per minute; *Salvia*, *S*.; Sample no., sample number; SEM, standard error of mean; SFDA, state food and drug administration; TCM, traditional Chinese medicine; THP, Taiwan herbal pharmacopeia; TLC, thin layer chromatography; TNF-α, tumor necrosis factor alpha; U.K., United Kingdom; U.S.A., United State of America; USP, United States pharmacopeia; UV, univariate scaling; VIP, variable importance; WHO, world health organization.
