# Supplementary material for: Danshen (Salvia miltiorrhiza) on the Global Market: What Are the Implications for Products’ Quality?
Source: Front Pharmacol. 2021 Apr 26;12:621169. doi: 10.3389/fphar.2021.621169 (PMC8107819; doi:10.3389/fphar.2021.621169)
Supplement: Supplementary file 1 [file DataSheet1.docx]

## Supplementary documents

**Tables**

[Suppl. table 1 Authenticated sample (A1 - A18) list](#_Toc62990567)

[Suppl. table 2 Chinese online store sample (C1 - C17) obtained via Taobao in 1st June 2017](#_Toc62990568)

[Suppl. table 3 Vietnamese sample (V1 - V15) list](#_Toc62990569)

[Suppl. table 4 Concentrated extract sample (E1 - E19) list](#_Toc62990570)

[Suppl. table 5 The chemical shift assignments of the chemical standards and its selected chemical shift linearity in the ^1^H NMR spectra](#_Toc62990571)

[Suppl. table 6 The physical properties of chemical standards (S1-S9)](#_Toc62990572)

[Suppl. table 7 Microwave digestion system program for metal analysis of Danshen samples and the parameters of the operation system of ICP-OES](#_Toc62990573)

[Suppl. table 8 Limitation of detection for each element in ICP-OES analysis](#_Toc62990574)

**Figures**

[*Suppl. figure 1 HPTLC results of the chemical standards (S1-S9) under 254nm with solvent system (toluene: chloroform: ethyl acetate: methanol: formic acid (v/v) = 2: 3: 4: 0.2: 2)*](#_Toc54259233)

[*Suppl. figure 2 ^1^H-NMR spectra of the chemical standards (S1-S9) from 10 ppm to 0.5 ppm and the Chemical shift assignment of the chemical standards in the ^1^H-NMR spectra of Danshen representative samples*](#_Toc54259234)

[*Suppl. figure 3 The ^1^H-NMR spectra of the Danshen samples*](#_Toc54259235)

[*Suppl. figure 4 The supplementary data of the PLS-DA of Chinese online store samples against Vietnamese samples*](#_Toc54259236)

[*Suppl. figure 5 The supplementary data of the PLS-DA of authenticated Salvia miltiorrhiza samples against Vietnamese samples*](#_Toc54259237)

[*Suppl. figure 6 The cell viability results of Danshen sample extracts in RAW 264.7 of all the samples at 100 ug/ml*](#_Toc54259238)

[*Suppl. figure 7 The effect of Danshen extracts on LPS induced NO production in RAW 264.7 of all the samples at 100 ug/ml*](#_Toc54259239)

| Sample No. | Name | Form | Originality | Collector | Collection time | Other information |
| --- | --- | --- | --- | --- | --- | --- |
| A1 | *Salvia miltiorrhiza* | Root | Pilot, VA, U.S.A | American herbal pharmacopeia | 30/08/2016 | Voucher specimen: #4331 |
| A2 | *Salvia miltiorrhiza* | Root | Williams, OR, U.S.A | American herbal pharmacopeia | 18/08/2016 | Voucher specimen: #4326, |
| A3 | *Salvia miltiorrhiza* | Root | Williams, OR, U.S.A | American herbal pharmacopeia | 27/03/2009 | Voucher specimen: #2592, |
| A4 | *Salvia miltiorrhiza* | Root | Jacksonville, OR, U.S.A | American herbal pharmacopeia | 25/08/2016 | Voucher specimen: #4320 |
| A5 | *Salvia miltiorrhiza* | Root | Petaluma, CA, U.S.A | American herbal pharmacopeia | 2007 | Voucher specimen: #2550 |
| A6 | *Salvia przewalskii* | Root | Petaluma, CA, U.S.A | American herbal pharmacopeia | 30/08/2016 | Voucher specimen: #4332 |
| A7 | *Salvia miltiorrhiza* | Root | Shandong, China | Brion | 23/09/2014 | Geno-verified Botanical Reference Material |
| A8 | *Salvia miltiorrhiza* | Root | Taiwan | YuFu biotek | 01/09/2016 | Voucher specimen: KKY 20160901, Oven 100 ̊C 30 min, 120 ̊C 30 min |
| A9 | *Salvia miltiorrhiza* | Root | Taiwan | YuFu biotek | 01/09/2016 | Voucher specimen: KKY 20160901, Oven 30 ̊C 48 hours |
| A10 | *Salvia miltiorrhiza* | Root | Taiwan | YuFu biotek | 01/09/2016 | Voucher specimen: KKY 20160901, Freeze dry 24 hours |
| A11 | *Salvia miltiorrhiza* | Root | Taiwan | YuFu biotek | 01/09/2016 | Voucher Specimen:  KKY 20160901  Oven 30 ̊C uncut 48 hours |
| A12 | *Salvia miltiorrhiza* | Root | Taiwan | YuFu biotek | 01/09/2016 | Voucher specimen: KKY 20160901, Air condition dry shade 48 hours |
| A13 | *Salvia miltiorrhiza* | Root | Germany | LfL experimental station Baumannshof | 10/2016 | Loamy sand (planting of seedlings gained from seeds, planting date April 2016). Dried at 42 °C with high air ventilation in a hurdle drier |
| A14 | *Salvia miltiorrhiza* | Root | Germany | LfL experimental station Baumannshof | 2014 | Planted in 2014 |
| A15 | *Salvia miltiorrhiza* | Root | China | NIFDC | Unknown | Governmental department raw material standard |
| A16 | *Salvia bowleyana* | Root | Jiangxi, China | Kew Gardens | 09-11/2001 | Voucher specimen: TCMK 226, EBC no.: 81195 |
| A17 | *Salvia przewalskii* | Root | China | Kew Gardens | 02/2018 | Voucher specimen: TCMK 790, EBC no.: 82956 |
| A18 | *Salvia miltiorrhiza* (wild) | Root | China | Kew Gardens | 11/2004 | Voucher specimen: TCMK391, EBC no.: 82956 |

Suppl. table 1 Authenticated sample (A1 - A18) list

| Sample No. | Commercial Name | Form | Originality | Quantity (g) | Retailer | Manufacturer | Cost in RMB | Cost in pound * | Cost per 100 g |
| --- | --- | --- | --- | --- | --- | --- | --- | --- | --- |
| C1 | Danshen (broken cell wall) | Granule | Shandong | 20 * 1 g | Jinkang Pharmacy Flagship Store 金康大药房旗舰店 | ZEUS 中智 | ¥ 60.00 | £ 6.84 | £ 34.20 |
| C2 | Danshen powder | Powder | Shandong | 2 * 150 g | Qiancaotang 千草堂 | Qiancaotang 千草堂 | ¥ 59.00 | £ 6.73 | £ 2.24 |
| C3 | Danshen powder | Powder | Anhui | 88 g | Kangmei 康美 | Kangmei Pharmaceutical Co., Ltd. | ¥ 38.80 | £ 4.42 | £ 5.03 |
| C4 | Danshen | Dried root | Shandong | 200 g | Beijing Tongrentang | Beijing Tongrentang | ¥ 32.00 | £ 3.65 | £ 1.82 |
| C5 | Danshen powder | Powder | Yunnan | 90 g | Qidan flagship store 七丹旗舰店 | Qidan 七丹 | ¥ 35.00 | £ 3.99 | £ 4.43 |
| C6 | Danshen powder | Powder | Juxian, Shandong | 100 g | Jinhao flagship store 金貅旗舰店 | Jinhao 金貅 | ¥ 15.80 | £ 1.80 | £ 1.80 |
| C7 | Purple Danshen powder | Powder | Diandong, Yunnan | 150 g | Noland Health Products Specialstores 诺兰德保健品专营店 | Zunrentang 尊仁堂 | ¥ 28.00 | £ 3.19 | £ 2.13 |
| C8 | Yimeng mountain special grade wild Danshen | Dried root | Yimeng Mountain, Shandong | 500 g | 80s farmer store 八零后农民小店 | / | ¥ 21.90 | £ 2.50 | £ 0.50 |
| C9 | Danshen powder | Powder | Qiubei County, Wenshan Prefecture, Yunnan | 250 g | Wenshan University Student Entrepreneurship Dream Shop  文山大学生创业梦之店 | / | ¥ 26.00 | £ 2.96 | £ 1.19 |
| C10 | Danshen powder | Powder | Yunnan | 180 g | Xiyi herbs 希夷药材 | / | ¥ 19.00 | £ 2.17 | £ 1.20 |
| C11 | Danshen | Dried root | Sichuan Zhongjiang | 500 g | Mr. Shiso  佰草氏 | / | ¥ 25.00 | £ 2.85 | £ 0.57 |
| C12 | Danshen | Dried root | Sichuan | 120 g | Leiyun Upper West District Pharmacy Flagship Store 雷允上西区大药房旗舰店 | Shanghai Kangqiao Chinese Medicine Co., Ltd.  上海康桥中药饮片有限公司 | ¥ 20.00 | £ 2.28 | £ 1.90 |
| C13 | Danshen | Dried root | Shandong | 250 g | Bohaixiang晟海祥 | Jiangsu Bohaixiang Pharmaceutical Co., Ltd. 江苏晟海祥药业有限公司 | ¥ 28.80 | £ 3.28 | £ 1.31 |
| C14 | Danshen | Dried root | Jiangsu | 100 g | Li Liangji Flagship Store 李良济旗舰店 | Suzhou Tianling Chinese Medicine Co., Ltd. 苏州市天灵中药饮片有限公司 | ¥ 17.00 | £ 1.94 | £ 1.94 |
| C15 | Danshen | Dried root | Sichuan Zhongjiang | 500 g | Yichitang TCM Pharmacy  一致堂中药材行 | / | ¥ 23.00 | £ 2.62 | £ 0.52 |
| C16 | Danshen | Dried root | Sichuan Zhongjiang | 250 g | 山男经方缘中药材保健养生精 | / | ¥ 25.00 | £ 2.85 | £ 1.14 |
| C17 | Danshen powder | Powder | Yunnan | 220 g | Shengshi Hanfang 盛世汉方 | Yunnan Manlei Trading Co., Ltd. 云南曼雷商贸有限公司 | ¥ 102.00 | £ 11.63 | £ 5.28 |
| * 1st June, 2017, Chinese Yuan exchange rate to British pound = 0.11399 according to www.xe.com  *All the Chinese was translated to English by pinyin or the meaning of the words | | | | | | | | | |

Suppl. table 2 Chinese online store sample (C1 - C17) obtained via Taobao in 1st June 2017

| Sample No. | Commercial Name | Form | Originality | Quantity (g) | Market Channel | Retailer | Cost VND | Cost in pound | Cost per 100g |
| --- | --- | --- | --- | --- | --- | --- | --- | --- | --- |
| V1 | Đan Sâm | Dried root | China | 100 g | TCM pharmacy/store | Phố Lãn Ông 32 | VND 15,000.00 | £0.53 | £0.53 |
| V2 | Đan Sâm | Dried root | Viet Nam | 100 g | TCM pharmacy/store | Phố Lãn Ông 69A | VND 20,000.00 | £0.70 | £0.70 |
| V3 | Đan Sâm | Dried root | China (imported through Vietnamese Lạng Sơn province) | 100 g | TCM pharmacy/store | Phố Lãn Ông 28B | VND 15,000.00 | £0.53 | £0.53 |
| V4 | Đan Sâm | Dried root | N.A. | 100 g | TCM pharmacy/store | Phố Lãn Ông 2 | VND 30,000.00 | £1.06 | £1.06 |
| V5 | Đan Sâm | Dried root | China | 100 g | TCM pharmacy/store | Phố Lãn Ông 36 | VND 20,000.00 | £0.70 | £0.70 |
| V6 | Đan Sâm | Dried root | N.A. | 100 g | TCM pharmacy/store | Phố Lãn Ông 30 | VND 15,000.00 | £0.53 | £0.53 |
| V7 | Đan Sâm | Dried root | China | 100 g | TCM pharmacy/store | Phố Lãn Ông 24 | VND 15,000.00 | £0.53 | £0.53 |
| V8 | Đan Sâm | Dried root | China | 100 g | TCM pharmacy/store | Phố Lãn Ông 8 | VND 20,000.00 | £0.70 | £0.70 |
| V9 | Đan Sâm | Dried root | Viet Nam | 100 g | TCM pharmacy/store | Phố Lãn Ông 38 | VND 25,000.00 | £0.88 | £0.88 |
| V10 | Đan Sâm | Dried root | China | 100 g | TCM pharmacy/store | Phố Lãn Ông 48 | VND 20,000.00 | £0.70 | £0.70 |
| V11 | Đan Sâm | Dried root | China | 100 g | TCM pharmacy/store | Phố Lãn Ông 33 | VND 20,000.00 | £0.70 | £0.70 |
| V12 | Đan Sâm | Dried root | Viet Nam (highland areas such as Tam Đảo and plateau land, e.g. Ha Noi) | 100 g | TCM pharmacy/store | Purchased through the internet (caythuocnam.com.vn) | VND 25,000.00 | £0.88 | £0.88 |
| V13 | Đan Sâm | Dried root | China | 100 g | TCM pharmacy/store | P310 nhà 7, Tập thể ĐH Thủy Lợi, F. Trung Liệt, Q. Đống Đa | VND 30,000.00 | £1.06 | £1.06 |
| V14 | Đan Sâm | Dried root | N.A. | 100 g | TCM pharmacy/store | N.A. | N.A | N.A | N.A |
| V15 | Đan Sâm | Dried root | N.A. | 100 g | TCM pharmacy/store | N.A. | N.A | N.A | N.A |
| * 27th July, 2016, Vietnamese Dong exchange rate to British pound = 0.0000339 according to www.xe.com | | | | | | | | | |

Suppl. table 3 Vietnamese sample (V1 - V15) list

| Extract No. | Commercial Name | Originality | Quantity (g) | Market | Market Channel | Retailer | Other information | Manufacturer | Cost in pound | Cost per 100 g |
| --- | --- | --- | --- | --- | --- | --- | --- | --- | --- | --- |
| E1 | Danshen Sheng Foong Extract Pulveres | Taiwan | 100 g | Hong Kong | Supplier | Sheng Foong Co., Ltd | GMP, contain starch and herb powder | Sheng Foong Co., Ltd | N.A. | N.A. |
| E2 | Chuan Danshen Extract | Taiwan | 100 g | Hong Kong | Supplier | Kaiser Pharmaceutical Co., Ltd | GMP | Kaiser Pharmaceutical Co., Ltd | N.A. | N.A. |
| E3 | Chuang Song Zong Dan Tsan Granula Subtilae | Taiwan | 100 g | Hong Kong | Supplier | Chuang Song-Zong Pharmaceutical Factory | GMP, contain starch and herb powder | Chuang Song-Zong Pharmaceutical Factory | N.A. | N.A. |
| E4 | *Salvia miltiorrhiza* Extract powder | Taiwan | 100 g | Hong Kong | Supplier | Sheng Chang Pharmaceutical Factory | GMP, contain starch and herb powder | Sheng Chang Pharmaceutical Factory | N.A. | N.A. |
| E5 | Tan shen Herbal extract | Taiwan | 100 g | Hong Kong | Supplier | Sun Ten Pharmaceutical Co., Ltd | GMP, contain starch and herb powder | Sun Ten Pharmaceutical Co., Ltd | N.A. | N.A. |
| E6 | Fu Fang Danshen Pian Herbal extract | Taiwan | 100 g | Hong Kong | Supplier | Sun Ten Pharmaceutical Co., Ltd | GMP, contain starch and herb powder | Sun Ten Pharmaceutical Co., Ltd | N.A. | N.A. |
| E7 | MinTong Danshen | Taiwan | 100 g | The U.K. | Internet | Vitamin World | GMP, contain starch and herb powder | Min Tong Pharmaceutical Co., Ltd | £ 24.19 | £ 24.19 |
| E8 | Danshen extract | China | 50 g | The U.K. | TCM pharmacy/store | 養元堂 | GMP | Yangyuantang - 養元堂 | £ 20.00 | £ 40.00 |
| E9 | 100 g concentrated powder | China | 100 g | The U.K. | Internet | Jiangyin Tianjiang Pharmaceutical Co., Ltd. | GMP, contain starch | Jiangyin Tianjiang Pharmaceutical Co., Ltd. | £ 13.97 | £ 13.97 |
| E10 | Danshen, Concentrated Powder | China | 100 g | The U.K. | supplier | Mayway | N.A. | Unknown | £ 8.00 | £ 8.00 |
| E11 | Salvia | China | 100 g | Hong Kong | TCM pharmacy/store | Skylight Pharmaceutical Co., Ltd | N.A. | Skylight Pharmaceutical Co., Ltd | N.A. | NA. |
| E12 | Herbal powder (Qin dynasty) | N.A. | 10 g | The U.K. | TCM pharmacy/store | Natural Health Acupuncture Massage Herbs | N.A. | Unknown | £ 15.00 | £ 150.00 |
| E13 | Danshen granule | China | 100 g | The U.K. | Supplier | Donica | GMP, Drug: Extract=1:10 | Beijing Temages Pharmaceutical Co., Ltd | £ 14.42 | £ 14.42 |
| E14 | Danshen  Concentrated Extract | Taiwan | 50 g | The U.K. | Supplier | Shizhen TCM UK Ltd. | GMP | Koda Pharmaceutical Co., Ltd | £ 20.00 | £ 40.00 |
| E15 | *Salvia miltiorrhiza* extract | Taiwan | 100 g | Hong Kong | TCM pharmacy/store | Han-Fang Chinese Medicine Co. Ltd. | GMP, contain starch | Han-Fang Chinese Medicine Co. Ltd. | £ 18.93 | £ 18.93 |
| E16 | *Salvia miltiorrhiza* extract | China | 100 g | Hong Kong | TCM pharmacy/store | Premier Concentrated Chinese Herbs (Hong Kong) | N.A. | Premier Concentrated Chinese Herbs (Hong Kong) | £ 10.60 | £ 10.60 |
| E17 | *Salvia miltiorrhiza* extract | Shandong, China | 200 * 2 g | Hong Kong | TCM pharmacy/store | Sanjiu Medical & Pharmaceutical Co., Ltd | N.A. | Sanjiu Medical & Pharmaceutical Co., Ltd | £ 37.86 | £ 9.47 |
| E18 | *Salvia miltiorrhiza* extract | China | 200 g | Hong Kong | TCM pharmacy/store | PuraPharm International (H.K.) Ltd | GMP | PuraPharm International (H.K.) Ltd | £ 32.56 | £ 16.28 |
| E19 | Dan Shen Salvia extract | Taiwan | 100 g | The U.K. | Internet | dullmeat | GMP, contain starch and herb powder | Sun Ten Pharmaceutical Co., Ltd | £ 12.50 | £ 12.50 |

Suppl. table 4 Concentrated extract sample (E1 - E19) list

| Chemical name |  | Type | Shift | Integral | J's |
| --- | --- | --- | --- | --- | --- |
| Salvianolic acid B | A | d | 7.58 | 1 | 15.81 |
|  | B | d | 7.31 | 1 | 8.51 |
|  | C | d | 6.85 | 1 | 8.47 |
|  | D | d | 6.74 | 1 | 8.12 |
|  | E | dd | 6.69 | 2 | 2.22, 2.08 |
|  | F | d | 6.64 | 1 | 7.99 |
|  | G | d | 6.6 | 1 | 2.07 |
|  | H | dd | 6.56 | 2 | 2.13, 8.13 |
|  | I | dd | 6.54 | 1 | 2.07, 8.07 |
|  | J | m | 6.33 | 2 |  |
|  | K | d | 5.7 | 1 | 4.11 |
|  | L | td | 5.05 | 2 | 4.58, 7.84, 7.72 |
|  | M | d | 4.43 | 1 | 4.21 |
|  | N | dd | 2.98 | 1 | 4.54, 14.38 |
|  | O | m | 2.91 | 2 |  |
|  | P | dd | 2.85 | 1 | 8.30, 14.41 |
| Tanshinone IIA | A | d | 7.84 | 1 | 8.16 |
|  | B | s | 7.74 | 1 |  |
|  | C | d | 7.61 | 1 | 8.14 |
|  | D | t | 3.1 | 2 | 6.31, 6.31 |
|  | E | m | 1.75 | 3 |  |
|  | F | m | 1.64 | 4 |  |
|  | G | s | 1.31 | 6 |  |
| Cryptotanshinone | A | d | 7.86 | 1 | 8.15 |
|  | B | d | 7.54 | 1 | 8.1 |
|  | C | t | 4.96 | 1 | 9.54, 9.54 |
|  | D | dd | 4.43 | 1 | 6.14, 9.45 |
|  | E | dq | 3.52 | 1 | 6.60, 6.60, 6.52, 9.69 |
|  | F | t | 3.11 | 2 | 6.38, 6.38 |
|  | G | m | 1.75 | 2 |  |
|  | H | m | 1.64 | 2 |  |
|  | I | d | 1.31 | 6 | 2.89 |
|  | J | d | 1.27 | 3 | 6.82 |
| Salvianolic acid A | A | d | 7.86 | 1 | 15.73 |
|  | B | d | 7.15 | 1 | 8.43 |
|  | C | m | 7.03 | 2 |  |
|  | D | m | 6.79 | 2 |  |
|  | E | d | 6.75 | 1 | 8.06 |
|  | F | d | 6.67 | 1 | 2.09 |
|  | G | d | 6.61 | 1 | 8.02 |
|  | H | d | 6.55 | 1 | 16.29 |
|  | I | dd | 6.47 | 1 | 2.08, 8.06 |
|  | J | d | 6.27 | 1 | 15.74 |
|  | K | dd | 4.93 | 1 | 3.25, 9.84 |
|  | L | dd | 3.03 | 1 | 3.25, 14.50 |
|  | M | dd | 2.76 | 1 | 9.92, 14.33 |
| Danshensu | A | s | 6.67 | 1 |  |
|  | B | d | 6.61 | 1 | 7.65 |
|  | C | d | 6.48 | 1 | 7.88 |
|  | D | d | 2.87 | 1 | 11.14 |
| Rosmarinic acid | A | d | 7.49 | 1 | 15.84 |
|  | B | d | 7.09 | 1 | 2.1 |
|  | C | dd | 7.03 | 1 | 2.12, 8.21 |
|  | D | d | 6.8 | 1 | 8.13 |
|  | E | d | 6.71 | 1 | 2.07 |
|  | F | d | 6.67 | 1 | 8.01 |
|  | G | dd | 6.56 | 1 | 2.07, 8.10 |
|  | H | d | 6.28 | 1 | 15.87 |
|  | I | dd | 5.05 | 1 | 4.26, 8.50 |
|  | J | dd | 3.01 | 1 | 4.24, 14.36 |
|  | K | dd | 2.93 | 1 | 8.49, 14.38 |
| Tanshinone I | A | d | 9.2 | 1 | 8.79 |
|  | B | dd | 8.49 | 1 | 0.91, 8.72 |
|  | C | d | 7.92 | 1 | 8.72 |
|  | D | d | 7.84 | 1 | 1.27 |
|  | E | dd | 7.64 | 1 | 6.93, 8.86 |
|  | F | d | 7.48 | 1 | 6.95 |
|  | G | s | 2.71 | 3 |  |
|  | H | d | 2.23 | 3 | 1.44 |
| Dihydrotanshinone I | A | d | 9.18 | 1 | 8.75 |
|  | B | dd | 8.5 | 1 | 8.76, 12.46 |
|  | C | d | 7.84 | 1 | 8.71 |
|  | D | dd | 7.67 | 1 | 6.91, 8.89 |
|  | E | d | 7.54 | 1 | 6.96 |
|  | F | t | 5.05 | 1 | 9.59, 9.59 |
|  | G | dd | 4.5 | 1 | 6.41, 9.41 |
|  | H | s | 2.72 | 3 |  |
|  | I | d | 2.69 | 1 | 17.17 |
|  | J | d | 1.32 | 3 | 6.83 |
| Caffeic acid | A | d | 7.43 | 1 | 15.84 |
|  | B | d | 7.05 | 1 | 2.12 |
|  | C | dd | 6.98 | 1 | 2.11, 8.19 |
|  | D | d | 6.78 | 1 | 8.15 |
|  | E | d | 6.2 | 1 | 15.84 |

Suppl. table 5 The chemical shift assignments of the chemical standards and its selected chemical shift linearity in the ^1^H NMR spectra

*All the chemicals standards were dissolved in DMSO_d6_ with 0.1% DSS as the internal standard reference and analysed in 500M Hz ­^1^H-NMR spectrometry. The structure ecluidation was based on the shape of the chemical shifts, the coupling of J’s magnitude in the ^1^H-NMR and literatures*

|  | Chemical Name | Molecule formula | CAS No. | Color in DMSO | R_f_ | Solubility in DMSO (mg/ml) | Molecular Weight (g/mol) |
| --- | --- | --- | --- | --- | --- | --- | --- |
| S1 | Salvianolic acid B | C_36_H_30_O_16_ | 121521-90-2 | Colorless | 0.19 | 100 | 718.61 |
| S2 | Tanshinone IIA | C_19_H_18_O_3_ | 568-72-9 | Red | 0.69 | 25 | 294.34 |
| S3 | Cryptotanshinone | C_19_H_20_O_3_ | 35825-57-1 | Orange | 0.61 | 12 | 296.36 |
| S4 | Salvianolic acid A | C_26_H_22_O_10_ | 96574-01-5 | Pale yellow | 0.24 | 125 | 494.5 |
| S5 | Danshensu | C_9_H_10_O_5_ | 76822-21-4 | Colorless | 0.21 | 44 | 198.174 |
| S6 | Rosmarinic Acid | ‎C_18_H_16_O_8_ | 20283-92-5 | Colorless | 0.26 | 25 | 360.318 |
| S7 | Tanshinone I | C_18_H_12_O_3_ | 568-73-0 | Red | 0.68 | 23 | 276.291 |
| S8 | Dihydrotanshinone | C_18_H_14_O_3_ | 87205-99-0 | Orange | 0.61 | 5 | 278.3 |
| S9 | Caffeic acid | C_9_H_8_O_4_ | 331-39-5 | Colorless | 0.49 | 7 | 180.16 |
| * The solubility was based on the datasheet from the corresponding chemical supplier | | | | | | | |

Suppl. table 6 The physical properties of chemical standards (S1-S9)

| Time (min) | Temperature (°C) |  | Operation | Parameters |
| --- | --- | --- | --- | --- |
| 0 → 10 | room temperature → 175 |  | Nebuliser | Concentric |
| 10 → 45 | 175 |  | Spray chamber | Cyclonic |
| 45 → 45 | 175 → 185 |  | Radio frequency power | 1.45 kW |
| 45 → 50 | 185 |  | Plasma gas flow rate | 13 L/min |
| 50 → 60 | 185 → room temperature |  | Auxiliary gas flow rate | 1.2 L/min |
|  |  |  | Nebulizer gas flow rate | 0.75 L/min |
|  |  |  | Replicates per sample | 3 |

Suppl. table 7 Microwave digestion system program for metal analysis of Danshen samples and the parameters of the operation system of ICP-OES

| Element | Wavelength [nm] | Limit min. [ppm] | Limit min. [mg/kg] | Limit max. [ppm] | Limit max. [mg/kg] |
| --- | --- | --- | --- | --- | --- |
| As | 189.04 | 0.0001 | 0.0178 | 1.208 | 203.37 |
| As | 228.81 | 0.0001 | 0.0178 | 1.208 | 203.37 |
| Cd | 214.44 | 0.0001 | 0.0178 | 1.208 | 203.37 |
| Cd | 226.50 | 0.0001 | 0.0178 | 1.208 | 203.37 |
| Cu | 224.70 | 0.0001 | 0.0178 | 12 | 2020.20 |
| Cu | 324.75 | 0.0001 | 0.0178 | 23.96 | 4033.67 |
| Pb | 220.35 | 0.0001 | 0.0178 | 12 | 2020.20 |
| Pb | 405.78 | 0.0001 | 0.0178 | 12 | 2020.20 |

Suppl. table 8 Limitation of detection for each element in ICP-OES analysis


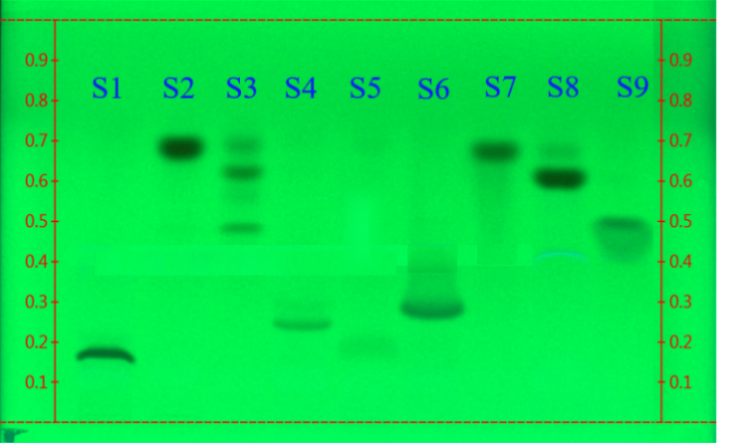


Suppl. figure 1 HPTLC results of the chemical standards (S1-S9) under 254nm with solvent system (toluene: chloroform: ethyl acetate: methanol: formic acid (v/v) = 2: 3: 4: 0.2: 2)

**
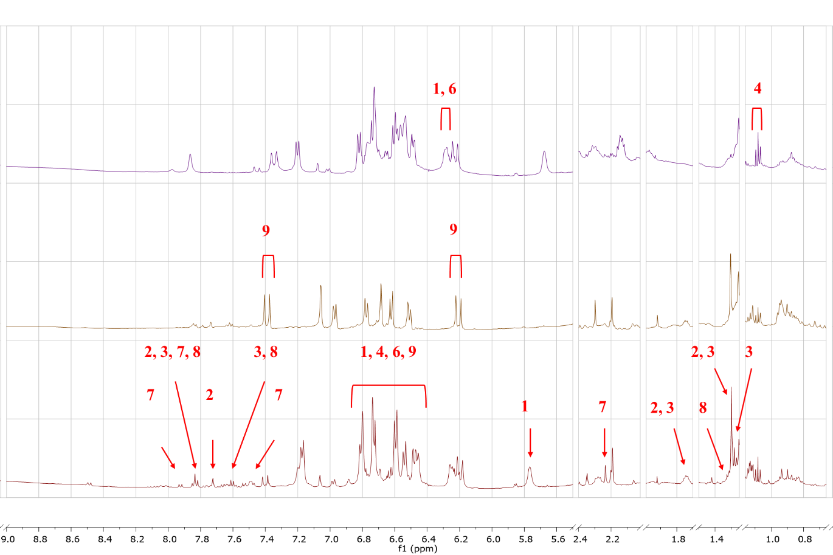
**


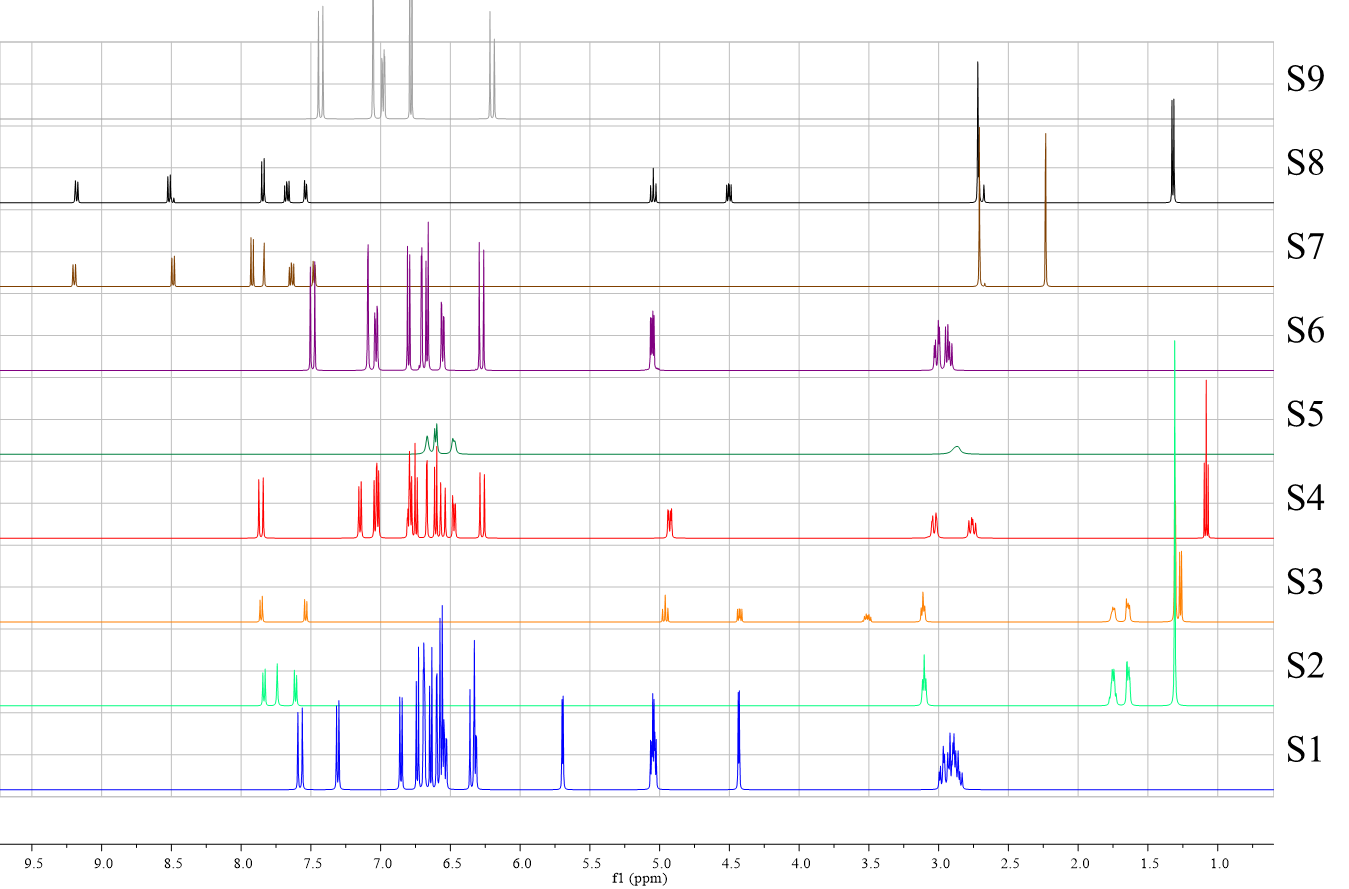


Suppl. figure 2 ^1^H-NMR spectra of the chemical standards (S1-S9) from 10 ppm to 0.5 ppm and the Chemical shift assignment of the chemical standards in the ^1^H-NMR spectra of Danshen representative samples

*All the chemicals standards (S1 = salvianolic acid B, S2 = tanshinone IIA, S3 = cryptotanshinone, S4 = salvianolic acid A, S5 = danshensu, S6 = rosmarinic acid, S7 = tanshinone I, S8 = dihydrotanshinone I and S9 = caffeic acid) were dissolved in DMSO_d6_ with 0.1% DSS as the internal standard reference and analysed in 500M Hz ­^1^H-NMR spectrometry. The spectra were modified to remove the noise, impurities and the solvent peaks*


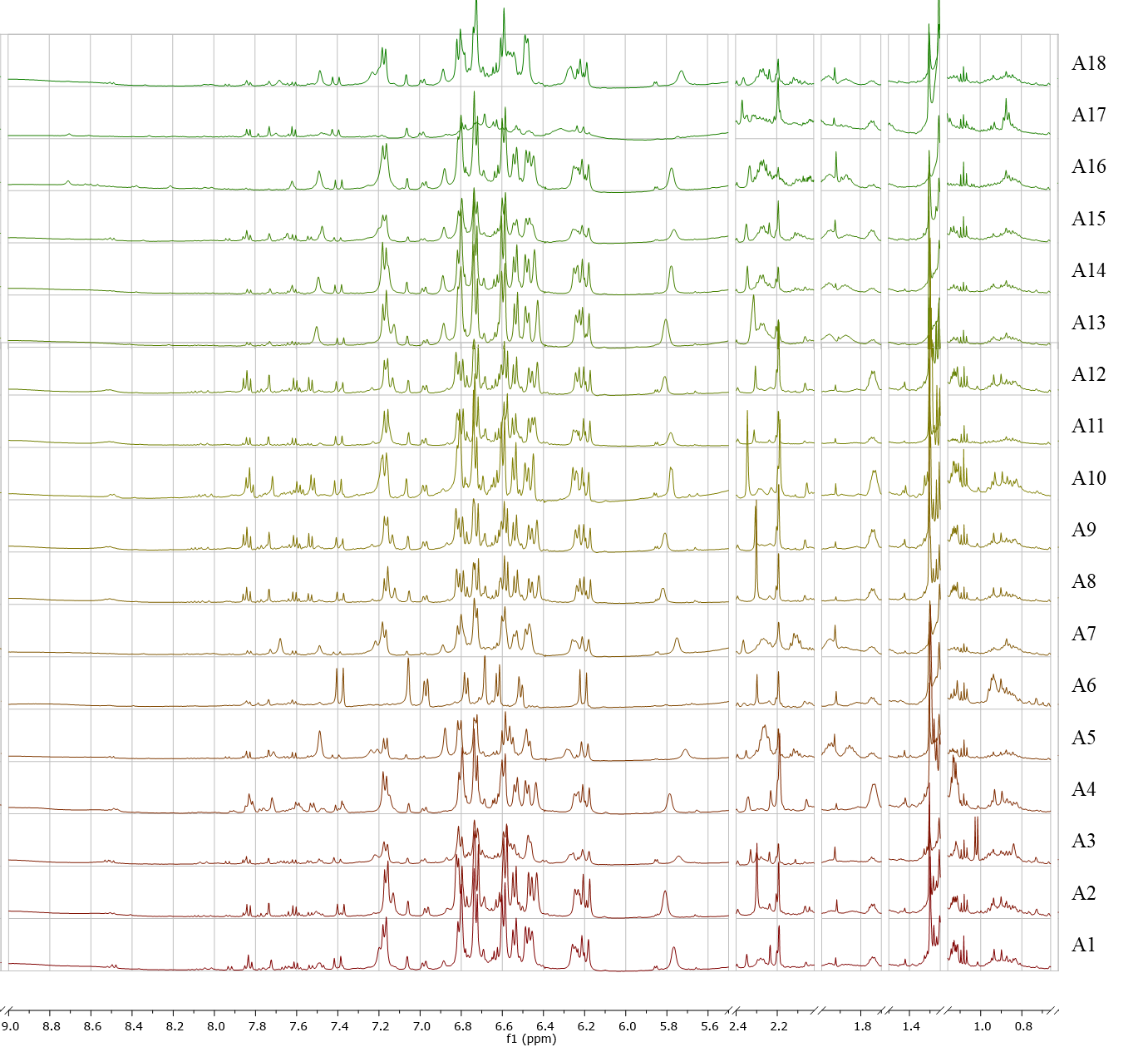


a)


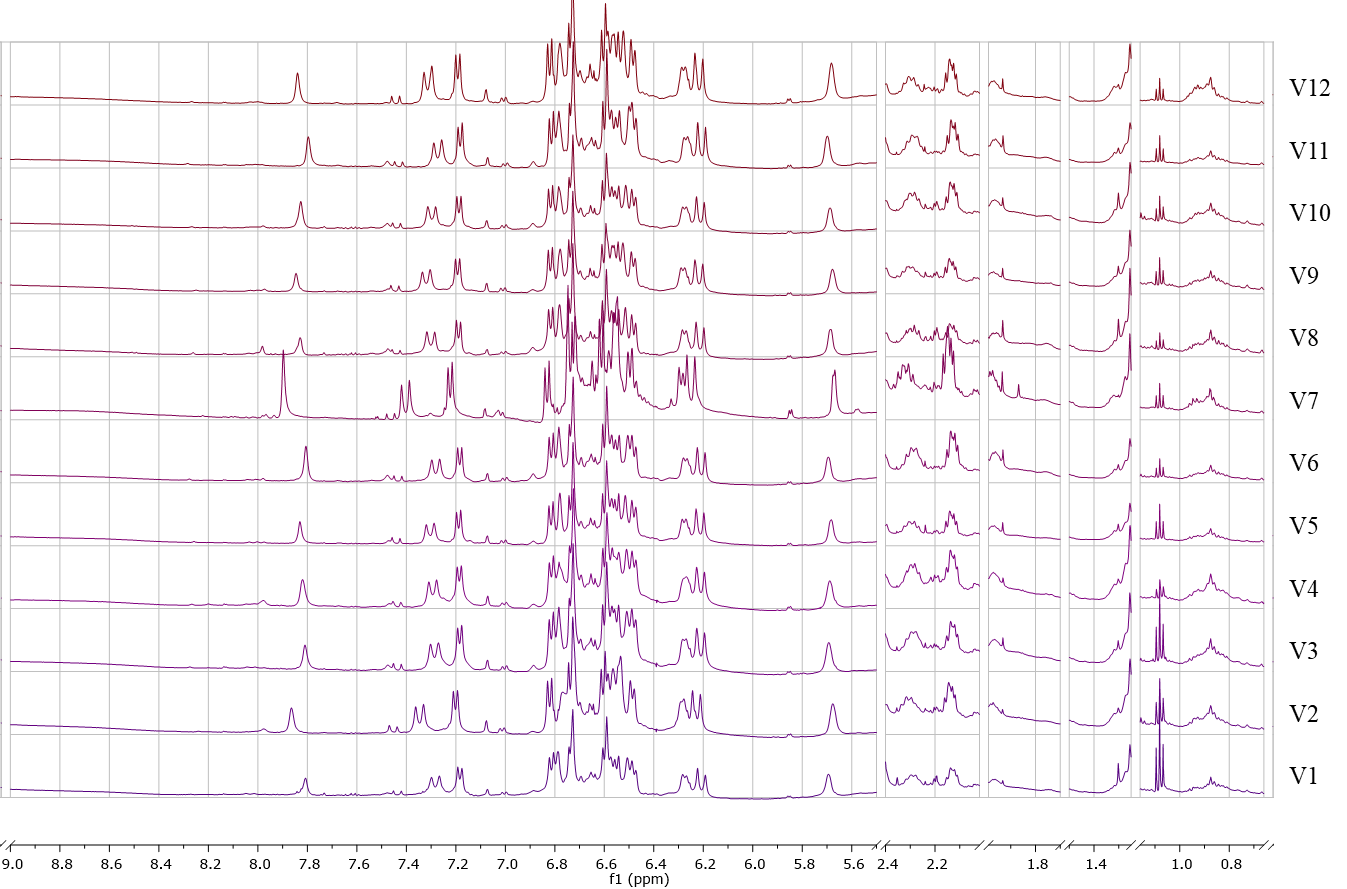


b)


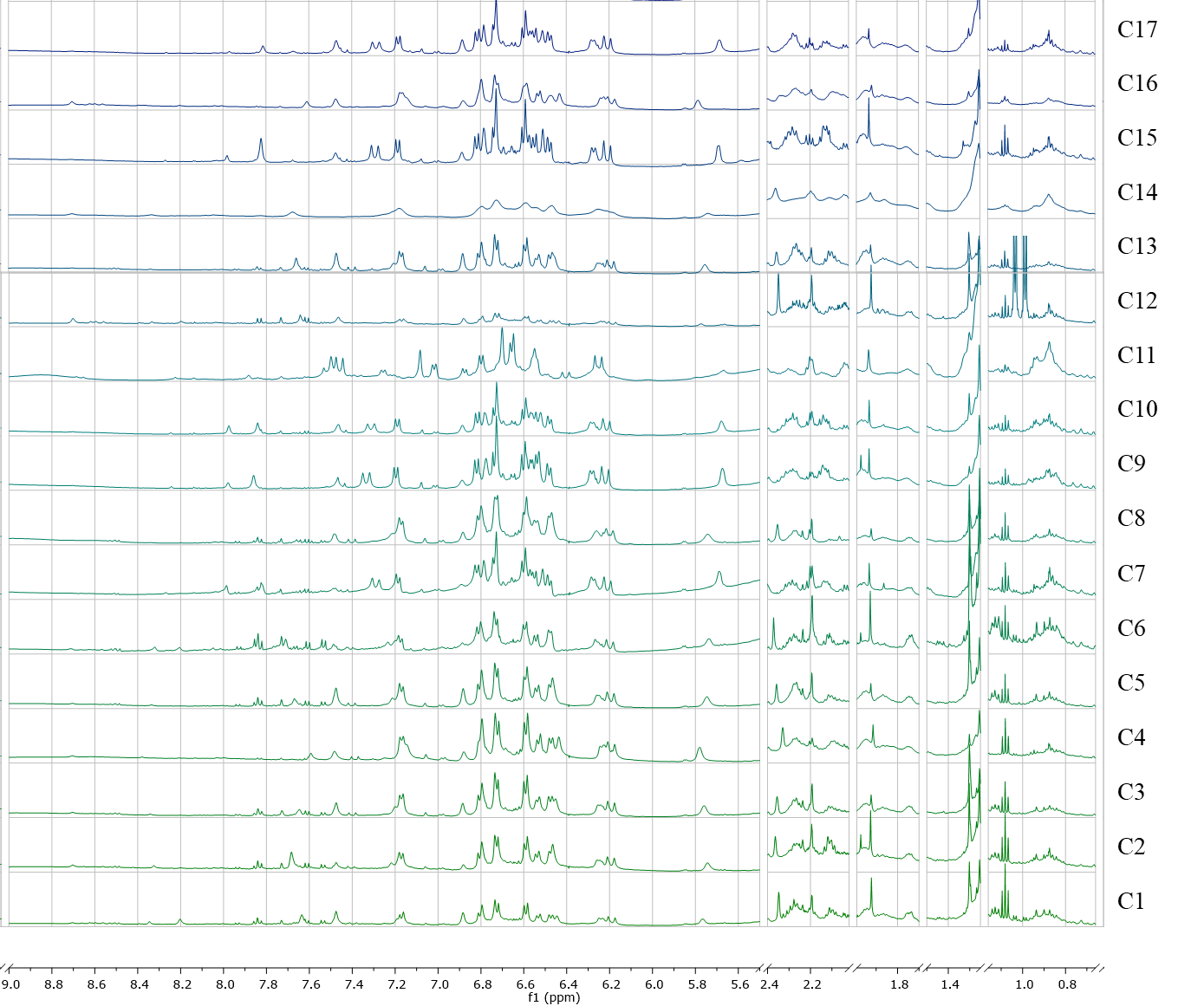


c)


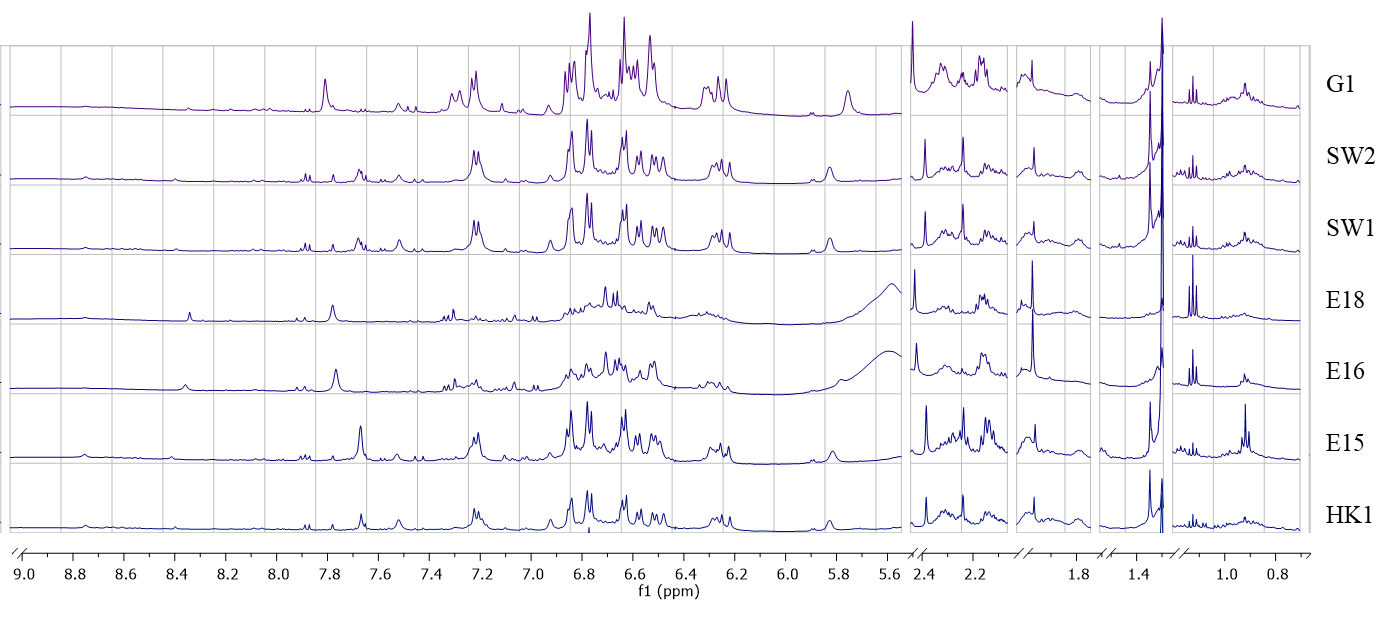


d)

Suppl. figure 3 The ^1^H-NMR spectra of the Danshen samples

*All the samples are extracted and processed at the same time. 0.8 g samples are sonicated for 30 minutes in4 75% methanol in sonication for 30 minutes and the extract is dried under 1.5 hour 60°C heating then freeze drying. The dried extract is redissolved DMSO_d6_ to 200 mg/ml. MestreNova 12.1 is used for the ^1^H-NMR spectra analysis. a) authenticated samples (A1 – A18) b) Vietnamese samples (V1 – V12) c) Chinese online store samples (C1 – C17) and d) individual samples and extracts (G1, SW1, SW2, HK1, and E15, E16 and E18)*


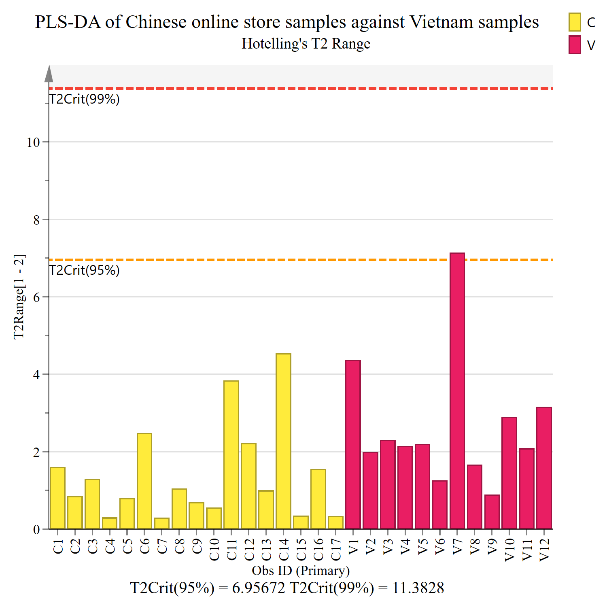

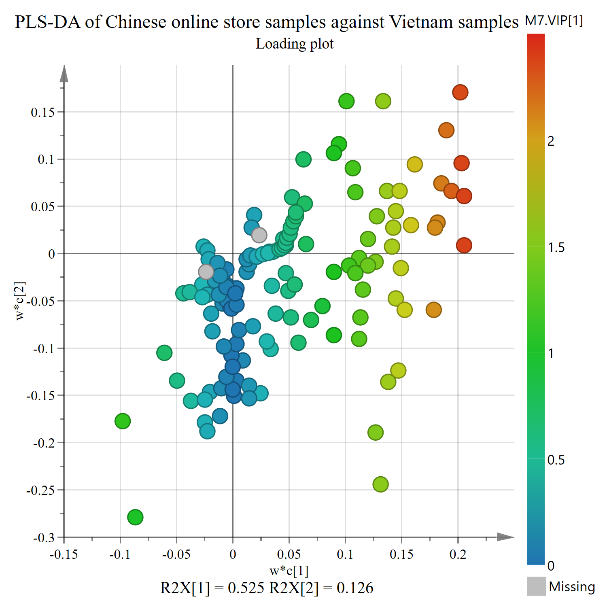


b)

a)


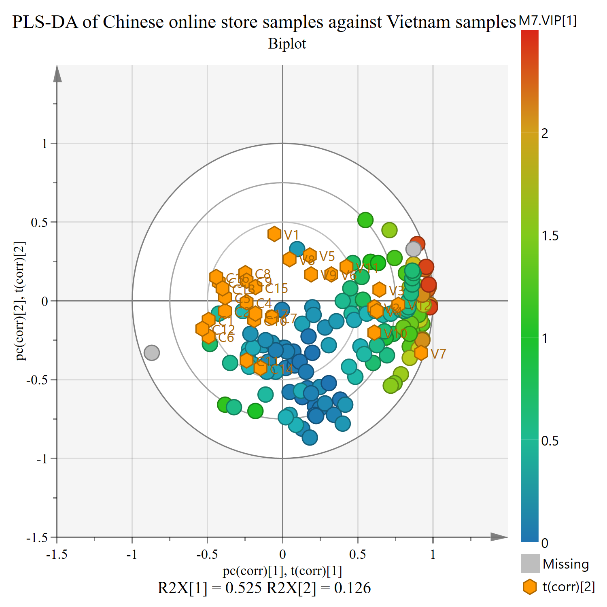

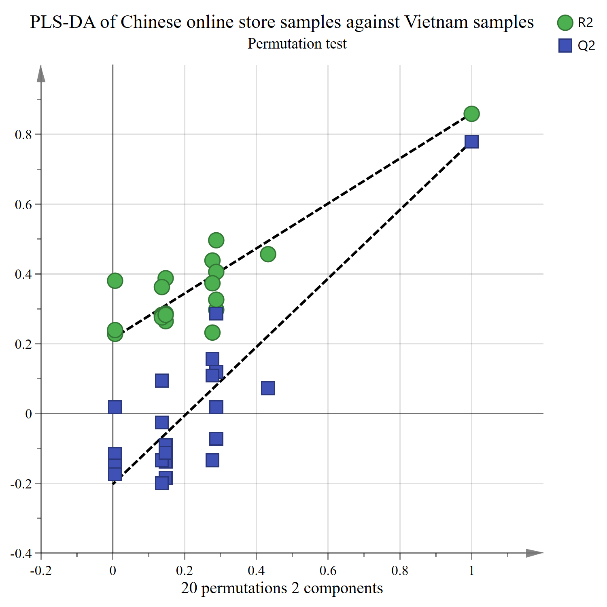


d)

c)

Suppl. figure 4 The supplementary data of the PLS-DA of Chinese online store samples against Vietnamese samples

*a) Hotelling T^2^ range b) coefficient plot, and c) biplot were referring the VIP value of the X variable contribute to principal component 1. d) permutation test showed the intercepts of R^2^ = (0.0, 0.224), and Q^2^ = (0.0, -0.27)*


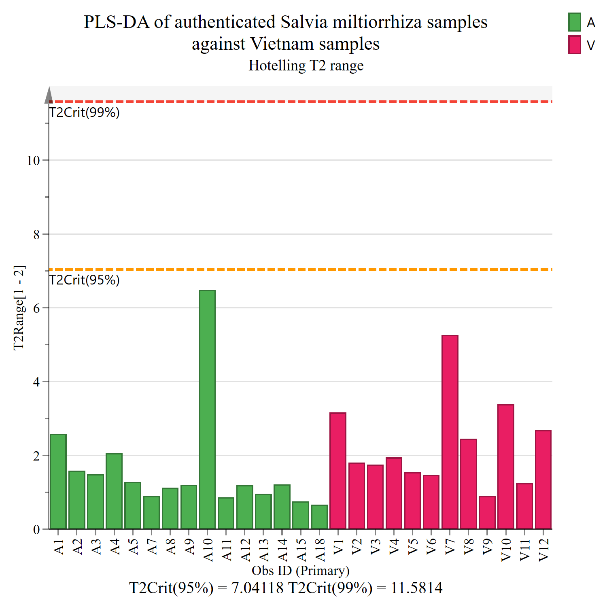

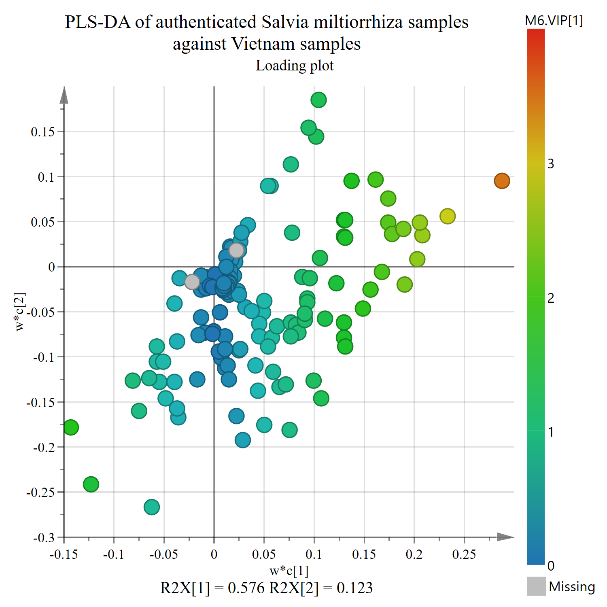


b)

a)


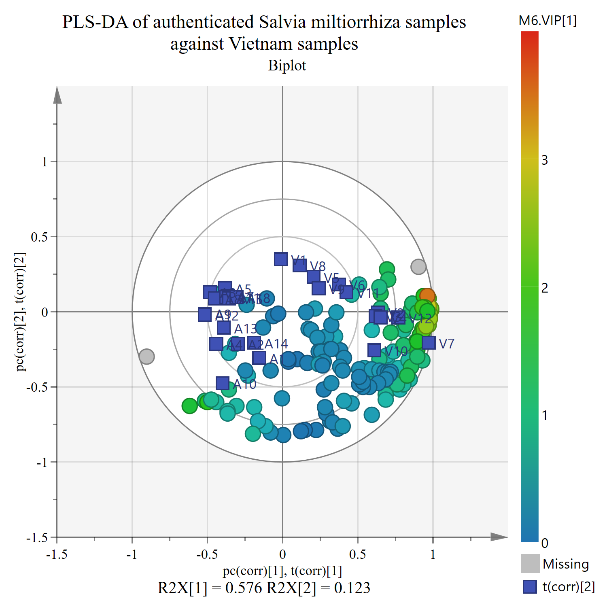

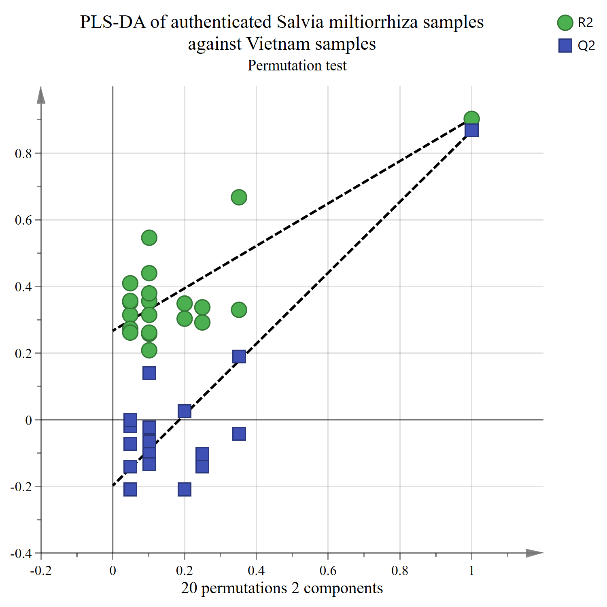


c)

d)

Suppl. figure 5 The supplementary data of the PLS-DA of authenticated Salvia miltiorrhiza samples against Vietnamese samples

*a) Hotelling T^2^ range b) coefficient plot, and c) biplot were referring the VIP value of the X variable contribute to principal component 1. d) permutation test showed the intercepts of R^2^ = (0.0, 0.267), and Q^2^ = (0.0, -0.197)*

Suppl. figure 6 The cell viability results of Danshen sample extracts in RAW 264.7 of all the samples at 100 ug/ml

Suppl. figure 7 The effect of Danshen extracts on LPS induced NO production in RAW 264.7 of all the samples at 100 ug/ml
